# Novel zebrafish polycystic kidney disease models reveal functions of the Hippo pathway in renal cystogenesis

**DOI:** 10.1242/dmm.049027

**Published:** 2021-11-09

**Authors:** Zhiqin Ren, Zhiwei Zhang, Tzu-Ming Liu, Wei Ge

**Affiliations:** Department of Biomedical Sciences and Centre of Reproduction, Development and Aging (CRDA), Faculty of Health Sciences, University of Macau, Taipa, Macau 999078, China

**Keywords:** Hippo signaling pathway, Polycystic kidney disease, Kidney development, Renal cyst formation, Zebrafish model

## Abstract

The Hippo signaling pathway is a kinase cascade that plays an important role in organ size control. As the main effectors of the Hippo pathway, transcription coactivators Yap1/Wwtr1 are regulated by the upstream kinase Stk3. Recent studies in mammals have implicated the Hippo pathway in kidney development and kidney diseases. To further illustrate its roles in vertebrate kidney, we generated a series of zebrafish mutants targeting *stk3*, *yap1* and *wwtr1* genes. The *stk3*^−/−^ mutant exhibited edema, formation of glomerular cysts and pronephric tubule dilation during the larval stage. Interestingly, disruption of *wwtr1*, but not *yap1*, significantly alleviated the renal phenotypes of the *stk3*^−/−^ mutant, and overexpression of Wwtr1 with the CMV promoter also induced pronephric phenotypes, similar to those of the *stk3*^−/−^ mutant, during larval stage. Notably, adult fish with Wwtr1 overexpression developed phenotypes similar to those of human polycystic kidney disease (PKD). Overall, our analyses revealed roles of Stk3 and Wwtr1 in renal cyst formation. Using a pharmacological approach, we further demonstrated that Stk3-deficient zebrafish could serve as a PKD model for drug development.

## Abstract (precis)

**Summary:** A zebrafish *stk3* mutant line and Wwtr1 overexpression line provide evidence for functions of the Hippo signaling pathway in renal cyst formation and represent potential models for polycystic kidney disease.

## INTRODUCTION

The Hippo signaling pathway is a kinase cascade first characterized in *Drosophila* for its role in controlling organ size (Harvey et al., 2003; Huang et al., 2005; Justice et al., 1995). Hippo (Hpo) can bind to scaffold protein Salvador (Sav) and phosphorylate Warts (Wts). The phosphorylated Wts then binds to Mob as tumor suppressor (Mats) and phosphorylates the downstream effector Yorkie (Yki) to inhibit its function as a transcriptional coactivator. Orthologs of all Hippo pathway components have been found in mammals. Mammalian Ste20-like kinases 1/2 (MST1/2, the orthologs of Hpo, also known as STK4/3) binds to WW domain-containing protein 1 (SAV1, the ortholog of Sav), a member of the scaffold protein Salvador family. Then, phosphorylated LATS1/2 bind to MOB kinase activator 1A/B (MOB1A/B, orthologs of Mats) and phosphorylate two downstream effectors, Yes-associated protein 1 (YAP1, the ortholog of Yki) and WW domain-containing transcription regulator protein 1 (WWTR1, paralog of YAP1, also known as TAZ) (Sansores-Garcia et al., 2011; Yu et al., 2013; Zhang et al., 2010). YAP1/WWTR1 are transcriptional coactivators as they do not bind to DNA and activate transcription directly. Instead, they interact with other transcription factors to regulate gene expression. When the Hippo signaling pathway is not activated, YAP1/WWTR1 are mainly retained in the nucleus and serve as transcription coactivators. When the pathway is activated, YAP1/WWTR1 are phosphorylated and translocate to the cytoplasm, losing their functions as transcription coactivators (Yu et al., 2015). TEA domain transcription factors (TEADs) are thought to be the most important partners of YAP1/WWTR1 (Zhao et al., 2008). YAP1/WWTR1 can also interact with other transcription factors, including SMADs, p63 (also known as TP63), RUNXs and PAXs (Varelas, 2014). Together with these transcription factors, YAP1/WWTR1 can regulate the expression of a variety of downstream genes related to cell survival, proliferation, differentiation and migration.

Recently, the Hippo signaling pathway was found to play potential roles in polycystic kidney diseases (PKDs), which are a group of inherited disorders characterized by cyst formation within the kidney (Cornec-Le Gall et al., 2019). Autosomal-dominant PKD (ADPKD) is the most common type of PKD, which is caused by mutation of the *PKD1* or *PKD2* gene (Hughes et al., 1995; Mochizuki et al., 1996). The symptoms of ADPKD are enlarged kidney and formation of multiple fluid-filled cysts (Wilson, 2004). Analysis of clinical samples from ADPKD patients showed that the Hippo signaling pathway was altered and YAP1/WWTR1 were activated in renal cysts (Cai et al., 2018; Happé et al., 2011). Further studies revealed that loss of YAP1/WWTR1 reduced cyst formation in a *Pkd1*-deficient mouse model (Cai et al., 2018).

In zebrafish, the orthologs of all Hippo signaling pathway components have also been identified. Some studies on function of the Hippo signaling pathway members in zebrafish have recently been reported based on gene-knockout techniques. A recent study reported that serine/threonine kinase 3 (Stk3, ortholog of MST1/MST2 in zebrafish) and Sav1 played essential roles in the zebrafish biliary system (Brandt et al., 2020). Yap1/Wwtr1 were together found to be important for embryonic development (Kimelman et al., 2017), in particular the development of liver (Yi et al., 2018), eye (Miesfeld et al., 2015) and blood vessels (Astone et al., 2018; Nakajima et al., 2017). Wwtr1 alone was essential for micropyle formation (Dingare et al., 2018; Yi et al., 2019) and cardiac wall maturation (Lai et al., 2018). Interestingly, dysfunction of the Hippo signaling pathway was also implicated in zebrafish renal cyst formation (He et al., 2015; Skouloudaki et al., 2009; Xu et al., 2018; Zhang et al., 2015). However, existing studies on renal cyst formation were fragmentary and mostly based on morpholino-mediated gene knockdown. Further investigation with gene knockout is required to provide more conclusive evidence.

In the past decades, the zebrafish has become a popular model for studying human diseases (Pickart and Klee, 2014), including renal cyst formation (Gehrig et al., 2018; Poureetezadi and Wingert, 2016). However, owing to the lethality of the phenotype, most studies have been confined to pronephric cyst formation during embryogenesis. As there are only two nephrons in zebrafish pronephros, a similar phenotype to human PKD cannot be found in zebrafish embryos. The lack of an adult model has limited the application of zebrafish in the study of PKD.

Here, we report the generation of a zebrafish Stk3-deficient mutant line and a Wwtr1 overexpression line followed by phenotype analysis. Our findings provided critical clues on the function of the Hippo signaling pathway in zebrafish renal cyst formation. Furthermore, with the generation of a novel mesonephric PKD model in zebrafish, we have established a connection between pronephric phenotype in larval stage and polycystic kidney phenotype in adult stage, demonstrating the potential value of the zebrafish *stk3* mutant and Wwtr1 overexpression line as models for PKD.

## RESULTS

### Global knockout of *stk3* in the zebrafish induces edema and causes larval lethality

**Fig. 1.**
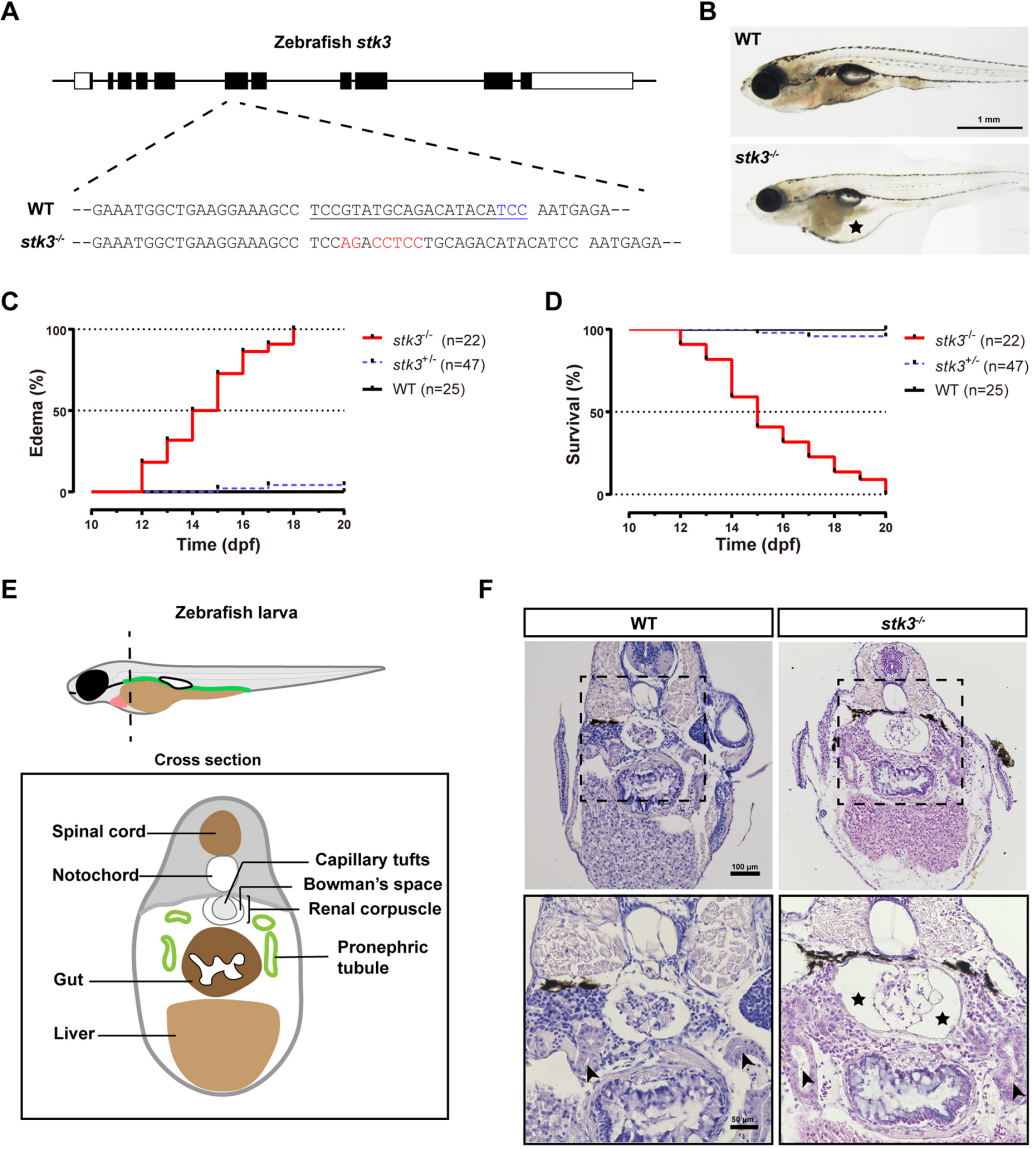
***stk3* mutation induced edema, glomerular cyst and pronephric tubule dilation in zebrafish.** (A) Schematic diagram of *stk3* gene structure and CRISPR/Cas9-induced mutation. The CRISPR site was designed to target the 6th exon. A mutant line with a 5-bp insertion was generated for phenotype analysis. Filled boxes indicate coding regions and open boxes indicate untranslated regions. The underlined sequence is the CRISPR site, the protospacer adjacent motif (PAM) sequence is shown in blue, and the altered sequence is shown in red. (B) Morphology of larvae at 15 dpf. The asterisk indicates the edema. Scale bar: 1 mm. (C) Kaplan–Meier plots for the onset of edema in *stk3*^−/−^ larvae and controls [wild-type (WT) and heterozygous siblings]. All the *stk3*^−/−^ larvae exhibited edema during 12-18 dpf, with median onset time at 14.5 dpf. Statistical analysis was performed using log-rank (Mantel–Cox) test (*P*<0.001). (D) Kaplan–Meier plots for survival of *stk3*^−/−^ larvae and controls (WT and heterozygous siblings). No *stk3*^−/−^ larvae could survive beyond 20 dpf, with median survival time at 15 dpf. Statistical analysis was performed using log-rank (Mantel–Cox) test (*P*<0.001). (E) Schematic diagram of larval cross section. (F) H&E staining of paraffin-embedded sections of *stk3*^−/−^ larvae and WT siblings at 15 dpf. The boxed areas are shown enlarged below. *stk3*^−/−^ larvae exhibited glomerular cysts with enlarged Bowman's space (asterisks) and pronephric tubule (arrowheads) dilation. Scale bars: 100 μm (top) and 50 μm (bottom).

To study the function of the Hippo signaling pathway in zebrafish, we applied the CRISPR/Cas9 system to target the 6th exon of the *stk3* gene (Fig. 1A). One mutant with a 5-bp insertion was generated, which resulted in nonsense frameshifting and a premature stop codon.

The homozygous *stk3* mutant (*stk3*^−/−^) was generated by in-crossing of the heterozygote (*stk3*^+/−^). All homozygous mutant larvae (*stk3*^−/−^) exhibited an edema phenotype of ascites during the period from 12 days post-fertilization (dpf) to 18 dpf (Fig. 1B). The Kaplan–Meier plot for edema onset showed that the median time of onset of edema was 14.5 dpf in the *stk3*^−/−^ mutant (Fig. 1C). After the onset of edema, the larvae died within 1 or 2 days. The survival curve showed that the median survival time was 15 dpf in the *stk3*^−/−^ mutant (Fig. 1D). To further investigate the phenotypes of the *stk3*^−/−^ mutant, we analyzed serial cross sections of the larvae at 15 dpf (Fig. 1E,F). Histological analysis revealed glomerular cyst formation and pronephric tubule dilation in the *stk3*^−/−^ larvae compared to the wild-type (WT) siblings. The areas of renal corpuscle in the *stk3*^−/−^ mutant were much larger than those in the WT siblings due to enlarged Bowman's spaces.

### Functional redundance of *yap1* and *wwtr1* in pronephros development at embryonic stage

To verify the role of the Hippo pathway in zebrafish renal development and understand its signaling mechanism, we further generated two mutant lines for zebrafish *yap1* and *wwtr1*, two downstream effectors of the Hippo pathway.

For *yap1* knockout, we targeted the 1st exon of *yap1* gene and obtained a mutant allele with a 4-bp deletion (*yap1*^−/−^) (Fig. S1A). The *yap1*^−/−^ mutant suffered from growth retardation during the juvenile stage but gradually caught up with *yap1*^+/+^ siblings at the adult stage (Fig. S1B-D). During development, *yap1*^−/−^ mutants did not exhibit edema or other kidney-related phenotypes. Histological analysis of adult *yap1*^−/−^ mutants showed no obvious dysfunction in the kidney compared to WT siblings (Fig. S1E).

For *wwtr1* knockout, we generated a zebrafish *wwtr1* mutant line by targeting the 1st exon of the gene. The mutant line carried a 7-bp deletion and a 293-bp insertion (Fig. S2A). Similar to the *yap1* mutant, the *wwtr1*^−/−^ mutants did not exhibit any signs of edema formation or other kidney-related phenotypes (Fig. S2B). Histological analysis of adult *wwtr1*^−/−^ mutants also showed no obvious dysfunction in the kidney (Fig. S2C).

Our data on *yap1*^−/−^ and *wwtr1*^−/−^ mutants differed from previous knockdown studies with morpholino (morphant), which showed that *yap1* and *wwtr1* both played roles in zebrafish pronephros development (He et al., 2015; Zhang et al., 2015). To further confirm this, we also performed an experiment to knock down the expression of *yap1* and *wwtr1* in F0 zebrafish embryos using a CRISPR/Cas9 approach (crispant). Different from the *yap1* knockout mutant (*yap1^−/−^*), the *yap1* crispant exhibited multiple defects, including pericardial edema, pronephric cysts and curved body (Fig. S3A). These phenotypes could be rescued with full-length *yap1* mRNA. Furthermore, *wwtr1* mRNA could also rescue the phenotypes of the *yap1* crispant, albeit to a lesser extent (Fig. S3B). Similarly, the *wwtr1* crispant exhibited multiple defects, including pronephric cysts and a curved body (Fig. S3C). These phenotypes could also be rescued with both *wwtr1* and *yap1* mRNA (Fig. S3D).

**Fig. 2.**
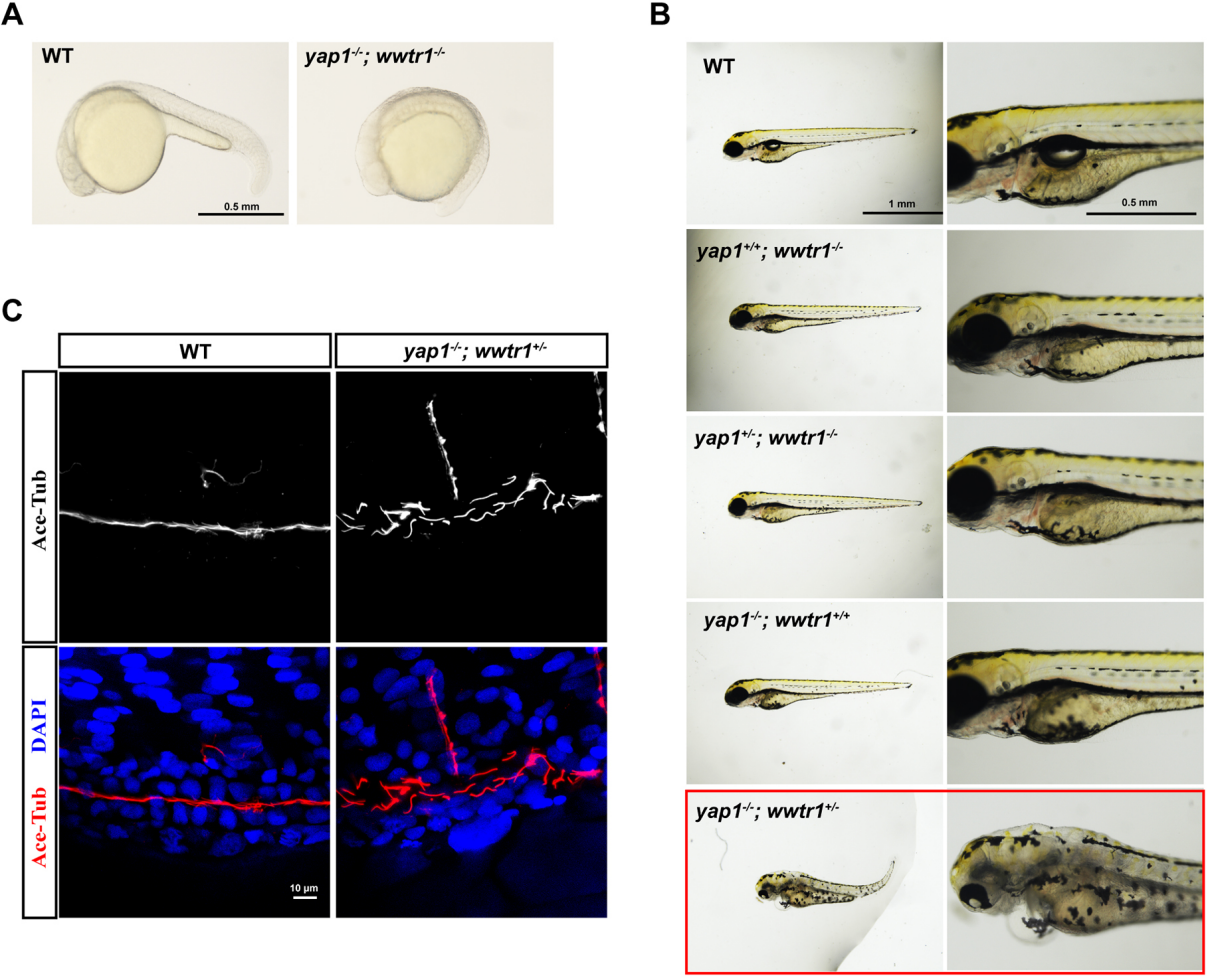
**Functional redundance of *yap1* and *wwtr1* in pronephros development at embryonic stage.** (A) Phenotype analysis of *yap1*^−/−^*;wwtr1*^−/−^ embryos at 20 hpf. The embryos of *yap1*^−/−^*;wwtr1*^−/−^ double mutant exhibited no tail extension. Scale bar: 0.5 mm. (B) Phenotype analysis of embryos with different genotypes produced by *yap1*^+/−^*;wwtr1*^+/−^ in-cross. Scale bars: 1 mm (left) and 0.5 mm (right). (C) Whole-mount immunofluorescent staining of acetylated tubulin (red) and DAPI (blue) in *yap1*^−/−^*;wwtr1*^+/−^ embryo and WT embryo at 48 hpf. *Z* projection of 0.5 μm optical section stacks. Scale bar: 10 μm.

The discrepancy between null mutants and crispants suggests potential functional compensation in the knockout, but not knockdown, mutants (El-Brolosy et al., 2019; Ma et al., 2019; Rossi et al., 2015). To confirm this hypothesis, we generated a *yap1* and *wwtr1* double mutant (*yap1*^−/−^*;wwtr1*^−/−^). As reported previously (Kimelman et al., 2017), the *yap1*^−/−^*;wwtr1*^−/−^ double mutants turned out to be embryonically lethal. The mutant embryos exhibited no tail extension and ceased development around 20 h post-fertilization (hpf) (Fig. 2A). The lethality could be rescued by *yap1* (*yap1*^+/+^*;wwtr1*^−/−^, *yap1*^+/−^*;wwtr1*^−/−^), as shown by normal embryonic development at 3 dpf. Interestingly, although *wwtr1* could also rescue the embryos, the restoration of normal development required both alleles of *wwtr1* (*yap1*^−/−^*;wwtr1*^+/+^). The *yap1*^−/−^*;wwtr1*^+/−^ fish exhibited multiple defects including pericardial edema, pronephric cysts and curved body, similar to those of the *yap1* and *wwtr1* crispants (Fig. 2B). We then performed whole-mount immunostaining with anti-acetylated tubulin (ace-Tub) to label cilia in pronephros, and maximal intensity projections of *z*-stack series were obtained to analyze the cilia. At 48 hpf, *yap1*^−/−^*;wwtr1*^+/−^ fish exhibited pronephric tubule dilation and abnormal cilia arrangement (Fig. 2C).

### The pronephric phenotypes in the *stk3*^−/−^ mutant are Yap1 independent

Stk3 is the core factor in the Hippo signaling pathway, which inhibits the activities of its downstream effectors Yap1/Wwtr1 through a phosphorylation cascade. When phosphorylated, Yap1/Wwtr1 translocate from the nucleus to the cytoplasm, therefore losing their functions as transcription coactivators (Yu et al., 2015). Disruption of *stk3* in the mutant could possibly result in the activation of Yap1/Wwtr1. To further understand the mechanisms of Stk3 action, we went on to demonstrate the involvement of Yap1/Wwtr1 in Stk3 signaling, and we examined Yap1 first.

**Fig. 3.**
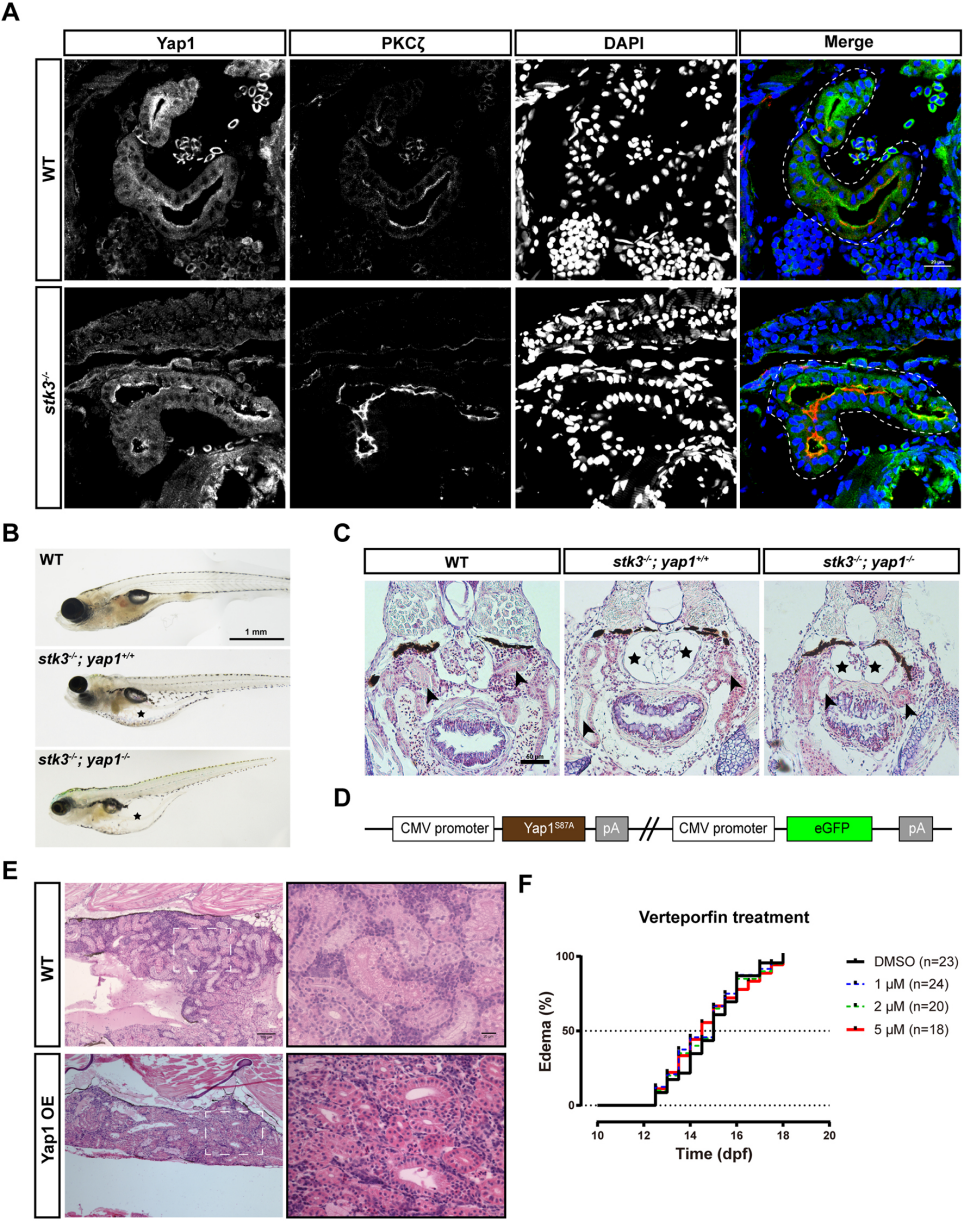
**Functional analysis of Yap1 in zebrafish *stk3*^−/−^ mutants.** (A) Immunofluorescent staining of Yap1 and PKCζ in cryostat sections of *stk3*^−/−^ larvae and WT siblings at 12 dpf. DAPI stains nuclei. The merged images are presented with Yap1 staining in green, PKCζ in red and DAPI in blue. Dashed line loops indicate the renal tubules. Scale bar: 20 μm. (B) Morphology of larvae at 15 dpf. Asterisks indicate the edema. Scale bar: 1 mm. (C) H&E staining of paraffin sections of larvae at 15 dpf. *stk3*^−/−^*;yap1*^−/−^ double mutants exhibited the same phenotype as *stk3*^−/−^ single mutants. Enlarged Bowman's space (asterisks) and pronephric tubule (arrowheads) dilation could be detected. Scale bar: 50 μm. (D) Schematic diagram of the expression plasmid for Yap1 OE fish line. (E) H&E staining of paraffin sections of adult kidneys at 90 dpf. No obvious difference between Yap1 OE fish and WT controls could be detected. Scale bars: 100 μm (left) and 20 μm (right). (F) Kaplan–Meier plots for the onset of edema in *stk3*^−/−^ larvae under verteporfin treatment. Treatment with a different dose of verteporfin (1, 2 and 5 μM) did not influence the onset of edema compared to DMSO control. Analysis with log-rank (Mantel–Cox) test showed no statistical significance.

The WT fish and *stk3*^−/−^ mutants at 12 dpf were sampled for cryostat sectioning and immunofluorescent staining with anti-YAP1 and anti-PKCζ antibodies. The anti-YAP1 antibody has been tested in a previous study (Brandt et al., 2019). PKCζ was used as a marker for apical regions of the renal tubules. In WT controls, Yap1 was mainly localized in the cytoplasm of tubular epithelial cells without showing nuclear accumulation (Fig. 3A). Surprisingly, there was no obvious difference in *stk3*^−/−^ mutants, suggesting that Yap1 was not activated. Then, we sampled the *stk3*^−/−^ mutants without edema at 15 dpf for the same analysis, and no obvious nuclear accumulation of Yap1 could be detected at this stage (Fig. S4A). These results indicated that activation of Yap1 was not the main cause of the pronephric phenotypes in *stk3*^−/−^ mutants and that Stk3-independent mechanisms exist to regulate the subcellular location of Yap1.

To confirm the above findings, we generated *stk3*^−/−^*;yap1*^−/−^ double mutants to study the function of Yap1 in *stk3*^−/−^ mutants. During larval stage, the *stk3*^−/−^*;yap1*^−/−^ double mutant also exhibited edema at the same time point as the *stk3*^−/−^ mutant (Fig. 3B). Histological analysis revealed that renal tubule dilation and glomerular cyst formation in the *stk3*^−/−^*;yap1*^−/−^ double mutant were comparable to those in the *stk3*^−/−^ mutant (Fig. 3C). Our observations suggested that the loss of Yap1 did not rescue or alleviate the pronephric phenotypes of the *stk3*^−/−^ mutant.

To further investigate the function of Yap1 in the zebrafish kidney, we overexpressed Yap1 in zebrafish. We cloned the coding sequence (CDS) of *yap1* from cDNA of zebrafish and introduced a mutation, which converted the 87th amino acid (conserved with mammalian YAP1 S127) from serine to alanine, which prevents potential phosphorylation. The expression of Yap1^S87A^ was driven by the cytomegalovirus (CMV) promoter (Fig. 3D). A stable transgenic line of *CMV:Yap1^S87A^* was generated with the Tol2 transposon system and we named it as Yap1 OE in this study. To confirm the functionality of Yap1 OE, we crossed this line with *yap1;wwtr1* double mutant. Yap1 OE was able to rescue the phenotypes of the *yap1*^−/−^*;wwtr1*^+/−^ fish (Fig. S4F). Before 5 dpf, ∼10-20% Yap1 OE embryos exhibited multiple defects, including ventral body curvature and pericardial edema (Fig. S4B,C), suggesting that Yap1 might have functions in early development. After removing these abnormal embryos at 5 dpf, the remaining fish were raised in the standard conditions, and no fish exhibited edema from larval stage to adult stage. Yap1 OE fish were identified through GFP signal (Fig. S4E). Histological analysis of adult fish showed no obvious dysfunction in the kidney of Yap1 OE fish (Fig. 3E).

To further confirm this, we tested the effects of verteporfin, an inhibitor of the YAP1-TEAD complex, on the *stk3*^−/−^ mutant. The progeny of *stk3*^+/−^ in-cross were treated with different doses of verteporfin (1, 2 and 5 μM). As *stk3*^−/−^ mutants normally exhibited edema between 12 dpf and 18 dpf, the treatment was initiated at 8 dpf and ended at 20 dpf. The results showed that verteporfin treatment did not influence the edema phenotype in *stk3*^−/−^ mutants (Fig. 3F).

Together, these results suggested that Yap1 was not activated in the renal tubular epithelial cells of *stk3*^−/−^ mutants and was therefore not responsible for the pronephric phenotypes of the *stk3*^−/−^ mutants.

### Activation of Wwtr1 contributes to the pronephric phenotypes of *stk3*^−/−^ mutant

**Fig. 4.**
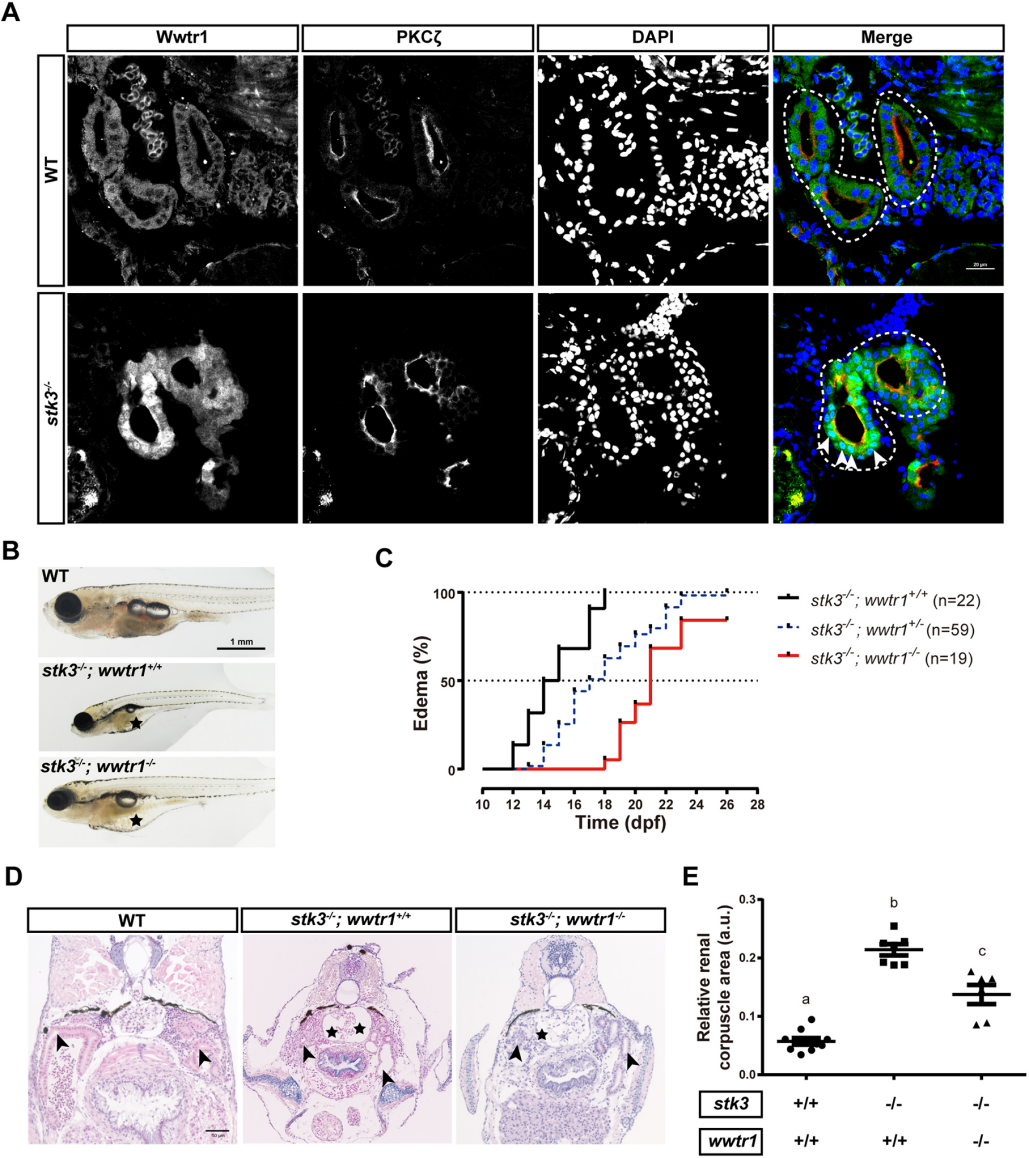
**The pronephric phenotypes of *stk3*^−/−^ mutants involve activation of Wwtr1.** (A) Immunofluorescent staining of Wwtr1, PKCζ and DAPI in cryostat sections of *stk3*^−/−^ larvae and WT siblings at 12 dpf. Merged images are presented with Wwtr1 staining in green, PKCζ in red and DAPI in blue. Dashed line loops indicate the renal tubules. The arrowheads indicate the nuclear accumulation of Wwtr1. Scale bar: 20 μm. (B) Morphology of larvae at 21 dpf. As no *stk3*^−/−^*;wwtr1*^+/+^ fish could be found at 21 dpf, we presented *stk3*^−/−^*;wwtr1*^+/+^ fish at 15 dpf for comparison. Asterisks indicate the edema. Scale bar: 1 mm. (C) Kaplan–Meier plot for the onset of edema in progeny of *stk3*^+/−^*;wwtr1*^+/−^ in-cross. *stk3*^−/−^*;wwtr1*^−/−^ double mutants exhibited delayed edema onset compared to *stk3*^−/−^ single mutants. Statistical analysis was performed using log-rank (Mantel–Cox) test (*P*<0.001). (D) H&E staining of paraffin sections of larvae at 21 dpf. *stk3*^−/−^*;wwtr1*^−/−^ double mutants exhibited mild glomerular cyst with enlarged Bowman's space (asterisks) and pronephric tubule (arrowheads) dilation. As no *stk3*^−/−^*;wwtr1*^+/+^ fish could be found at 21 dpf, we presented *stk3*^−/−^*;wwtr1*^+/+^ fish at 15 dpf for comparison. Scale bar: 50 μm. (E) Statistical analysis of relative renal corpuscle areas [renal corpuscle area/(trunk width)^2^]. *stk3*^−/−^*;wwtr1*^−/−^ double mutants exhibited significantly smaller renal corpuscles than *stk3*^−/−^ single mutants (*stk3*^−/−^*;wwtr1*^+/+^). Different letters indicate statistical significance by one-way ANOVA, mean±s.e.m. [*n*=10 *stk3*^+/+^*;wwtr1*^+/+^ (*n*=5 at 15 dpf, *n*=5 at 21 dpf), *n*=7 *stk3*^−/−^*;wwtr1*^+/+^ (15 dpf), *n*=6 *stk3*^−/−^*;wwtr1*^−/−^ (21 dpf); as the edema onset times were different in *stk3*^−/−^*;wwtr1*^+/+^ and *stk3*^−/−^*;wwtr1*^−/−^ lines, we sampled the fish at their median edema onset times]. a.u., arbitrary units.

After excluding the involvement of Yap1, we then investigated the activity of Wwtr1 in *stk3*^−/−^ mutants. We first performed immunostaining to examine the subcellular location of Wwtr1. The *stk3*^−/−^ mutants and WT siblings at 12 dpf were sampled for cryostat sectioning and immunofluorescent staining with anti-YAP/TAZ antibody labeling Wwtr1, anti-PKCζ antibody labeling apical regions of the renal tubules and DAPI labeling cell nuclei. The anti-YAP/TAZ antibody was confirmed to be specifically detecting Wwtr1 in zebrafish in a previous study (Kimelman et al., 2017). In WT controls, Wwtr1 was mainly localized in the cytoplasm of tubular epithelial cells, showing little nuclear accumulation (Fig. 4A). However, in *stk3*^−/−^ mutants, Wwtr1 was strongly localized in the nuclei of tubular epithelial cells, suggesting activation of Wwtr1 in *stk3*^−/−^ mutants.

Although Wwtr1 was activated in *stk3*^−/−^ mutants, we could not conclude that its activation was responsible for the pronephric phenotypes of the *stk3*^−/−^ mutant. To provide further evidence, we generated *stk3*^−/−^*;wwtr1*^−/−^ double mutants to study the involvement of Wwtr1 in the *stk3*^−/−^ mutants. During larval stage, the *stk3*^−/−^*;wwtr1*^−/−^ double mutants exhibited a significantly delayed onset of edema compared to *stk3*^−/−^ single mutants (Fig. 4B). The median times of onset of edema were 21 dpf in the *stk3*^−/−^*;wwtr1*^−/−^ double mutants, 17 dpf in the *stk3*^−/−^*;wwtr1*^+/−^ mutants and 14.5 dpf in the *stk3*^−/−^*;wwtr1*^+/+^ mutants, showing a clear dose-dependent pattern (Fig. 4C). Histological analysis of the *stk3*^−/−^*;wwtr1*^−/−^ double mutant at 21 dpf revealed mild renal tubule dilation and glomerular cyst formation (Fig. 4D). Quantification of the renal corpuscle areas showed significantly smaller renal corpuscles in *stk3*^−/−^*;wwtr1*^−/−^ double mutants than in *stk3*^−/−^*;wwtr1*^+/+^ single mutants (Fig. 4E). As the *yap1^−/−^;wwtr1^−/−^* double mutants exhibited severe defects during embryonic development (Fig. 2A), we were unable to obtain a *stk3^−/−^; yap1^−/−^;wwtr1^−/−^* triple mutant for further study*.*

Our observations suggested that the absence of Wwtr1 can significantly alleviate the renal abnormalities of *stk3*^−/−^ mutants, suggesting its involvement in the phenotypic development of the *stk3*^−/−^ mutants.

### Zebrafish with overexpression of Wwtr1 phenocopy *stk3*^−/−^ mutants at larval stage and develop PKD symptoms at adult stage

**Fig. 5.**
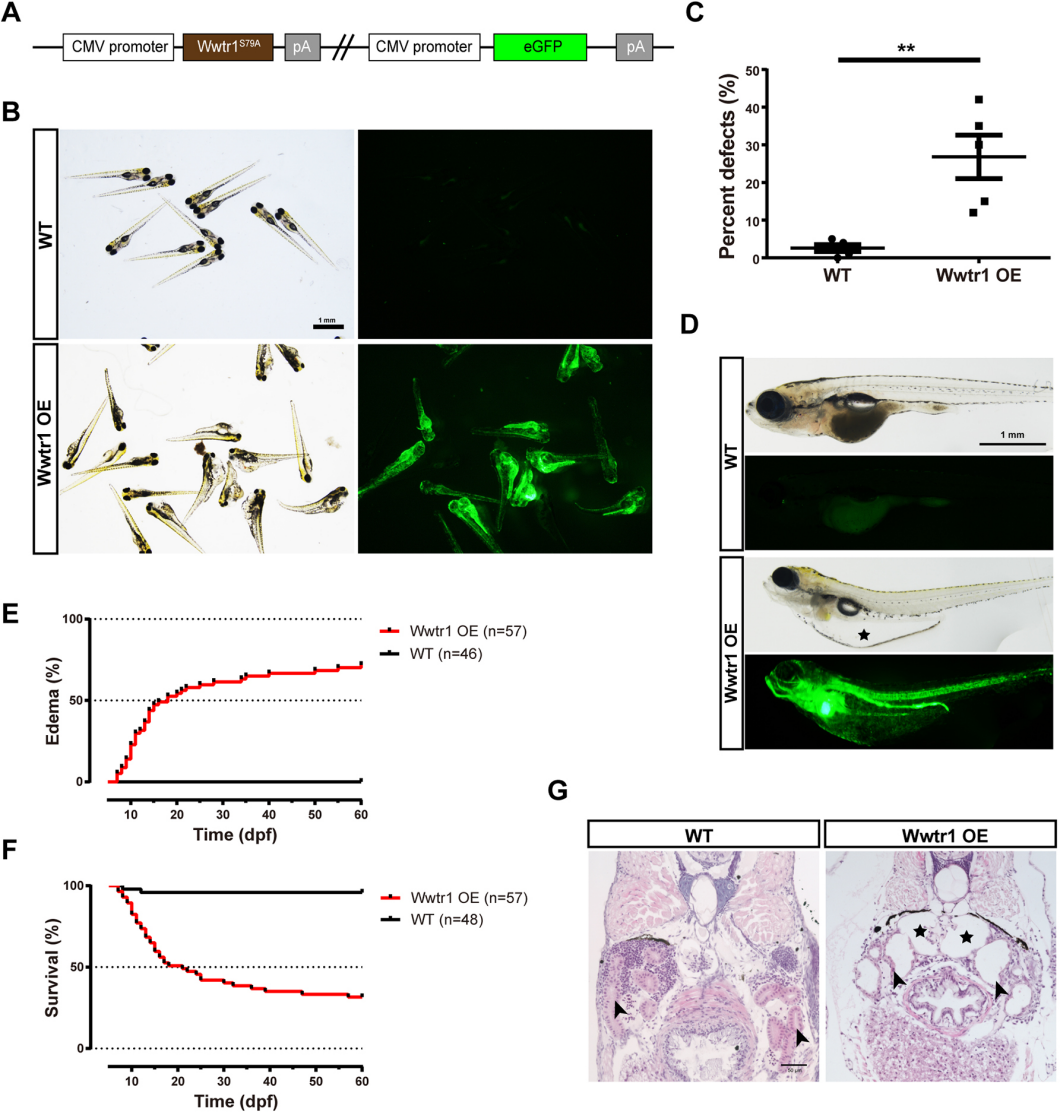
**Overexpression of Wwtr1 in zebrafish induces polycystic kidney disease (PKD).** (A) Schematic diagram of the expression plasmid for the Wwtr1 overexpression (Wwtr1 OE) fish line. (B) Morphology and fluorescent signal of larvae at 3 dpf. Scale bar: 1 mm. (C) Statistical analysis of embryonic defects in Wwtr1 OE fish and WT fish. **P<0.01 by unpaired two-tailed Student's *t*-test, mean±s.e.m. (*n*=5). (D) Morphology of larvae at 18 dpf. The asterisk indicates the edema. Scale bar: 1 mm. (E) Kaplan–Meier plots for the onset of edema in Wwtr1 OE and WT fish. The median time of onset of edema was 18 dpf. Statistical analysis was performed using log-rank (Mantel–Cox) test (*P*<0.001). (F) Kaplan–Meier plots for survival of Wwtr1 OE and WT fish. The median survival time was 21 dpf. Statistical analysis was performed using log-rank (Mantel–Cox) test (*P*<0.001). (G) H&E staining of paraffin sections of Wwtr1 OE and WT fish at 18 dpf. Wwtr1 OE fish exhibited glomerular cyst with enlarged Bowman's space (asterisks) and pronephric tubule (arrowheads) dilation. Scale bar: 50 μm.

To further investigate the function of Wwtr1 in zebrafish kidney development, we generated a transgenic line overexpressing constitutively active Wwtr1 (Wwtr1^S79A^). The CDS of *wwtr1* was cloned with a mutation introduced, which converted the 79th amino acid (conserved with mammalian WWTR1 S89) from serine to alanine to prevent potential phosphorylation. The expression of Wwtr1^S79A^ was driven by the CMV promoter (Fig. 5A), and a stable transgenic line of *CMV:Wwtr1^S79A^* was generated with the Tol2 transposon system, which we named Wwtr1 OE. To confirm the function of Wwtr1 OE, we crossed this line with the *yap1;wwtr1* double mutant. Wwtr1 OE could rescue the phenotype of the *yap1*^−/−^*;wwtr1*^+/−^ fish (Fig. S5A).

Before 5 dpf, ∼10-40% of Wwtr1 OE embryos exhibited multiple defects including ventral body curvature and pericardial edema (Fig. 5B,C). After removing these abnormal embryos at 5 dpf, the remaining fish were raised under standard conditions. During their development, some fish exhibited edema at different ages from larval to adult stage, with the median time of onset of edema being 18 dpf (Fig. 5D,E). The survival curve showed that the median survival time was 21 dpf (Fig. 5F). Some of the Wwtr1 OE fish could survive to adulthood.

During larval stage, the fish with edema were sampled for phenotype analysis. Hematoxylin and Eosin (H&E) staining revealed that Wwtr1 OE larvae exhibited renal tubule dilation and glomerular cyst formation with enlarged Bowman's space, which were nearly identical to the phenotypes of *stk3*^−/−^ mutants (Fig. 5G; Fig. S5C). These findings provided further evidence that the activation of Wwtr1 was responsible for the renal phenotypes in *stk3*^−/−^ mutants.

**Fig. 6.**
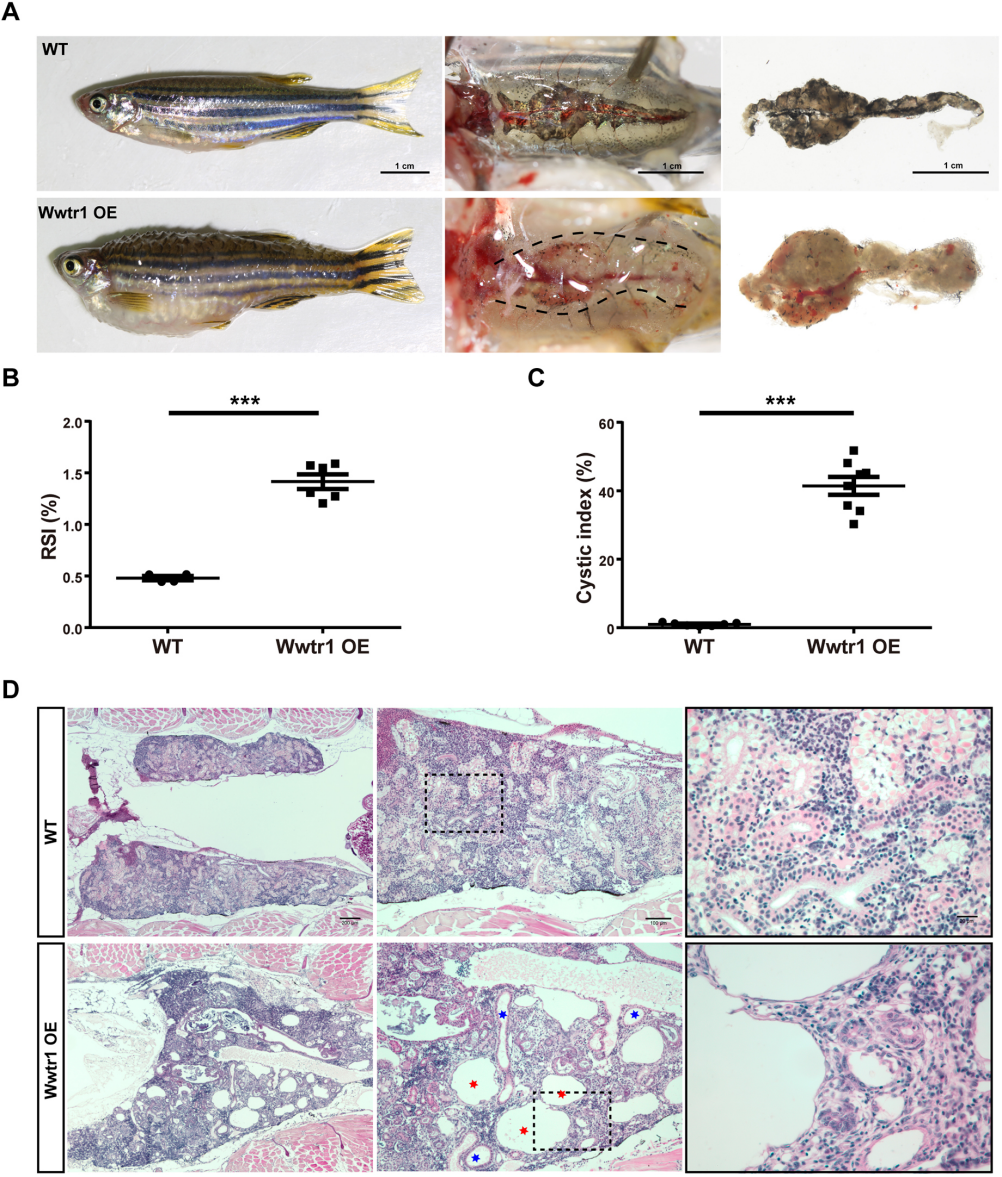
**Overexpression of Wwtr1 in zebrafish induces PKD (continued).** (A) Body and kidney morphology of adult fish at 90 dpf. Wwtr1 OE fish exhibited edema and abnormally larger kidneys compared with WT fish. Scale bar: 1 cm. (B) Statistical analysis of reno-somatic index (RSI; kidney weight/body weight) of Wwtr1 OE and WT fish (***P<0.001 by unpaired two-tailed Student's *t*-test, mean±s.e.m., *n*=6 WT, *n*=4 Wwtr1 OE). The Wwtr1 OE fish had significantly larger kidneys than WT fish. (C) Statistical analysis of cystic index (cystic area/total kidney area) of Wwtr1 OE and WT fish (***P<0.001 by unpaired two-tailed Student's *t*-test, mean±s.e.m., *n*=7 WT, *n*=8 Wwtr1 OE). (D) H&E staining of paraffin sections of adult kidneys at 90 dpf. Wwtr1 OE fish exhibited PKD. Red asterisks indicate glomerular cysts and blue asterisks indicate renal tubular cysts. The boxed areas are shown enlarged on the right. Scale bars: 200 μm (left), 100 μm (middle) and 20 μm (right).

Then, we further investigated the phenotypes in surviving adults. Wwtr1 OE fish were identified through GFP signal (Fig. S5D). Dissection of adult Wwtr1 OE fish revealed abnormal kidneys (Fig. 6A). The normal kidneys in WT fish are flattened organs attached tightly to the dorsal side of the body cavity and covered by abundant pigment cells (melanocytes). In comparison, the kidneys of Wwtr1 OE fish were much larger and thicker with bulges on the surface, making it much easier to separate them from the body wall. They also showed much fewer melanocytes (Fig. 6A). Reno-somatic index (RSI) analysis showed almost three times higher RSI in the Wwtr1 OE fish than in the WT control (Fig. 6B). Histological analysis revealed that Wwtr1 OE fish exhibited severe renal tubule dilation and massive cyst formation in the kidney, which is similar to the mammalian PKD phenotype (Fig. 6D; Fig. S6A,B). Quantitative analysis showed an average ∼40% cystic index in Wwtr1 OE fish, much higher than that in the WT control (Fig. 6C). Both glomerular cysts and renal tubular cysts could be detected (Fig. 6D). Thus, overexpression of active Wwtr1 induced cyst formation in both pronephros and mesonephros of zebrafish.

### Wwtr1 OE fish exhibit hyperproliferation in the kidney

**Fig. 7.**
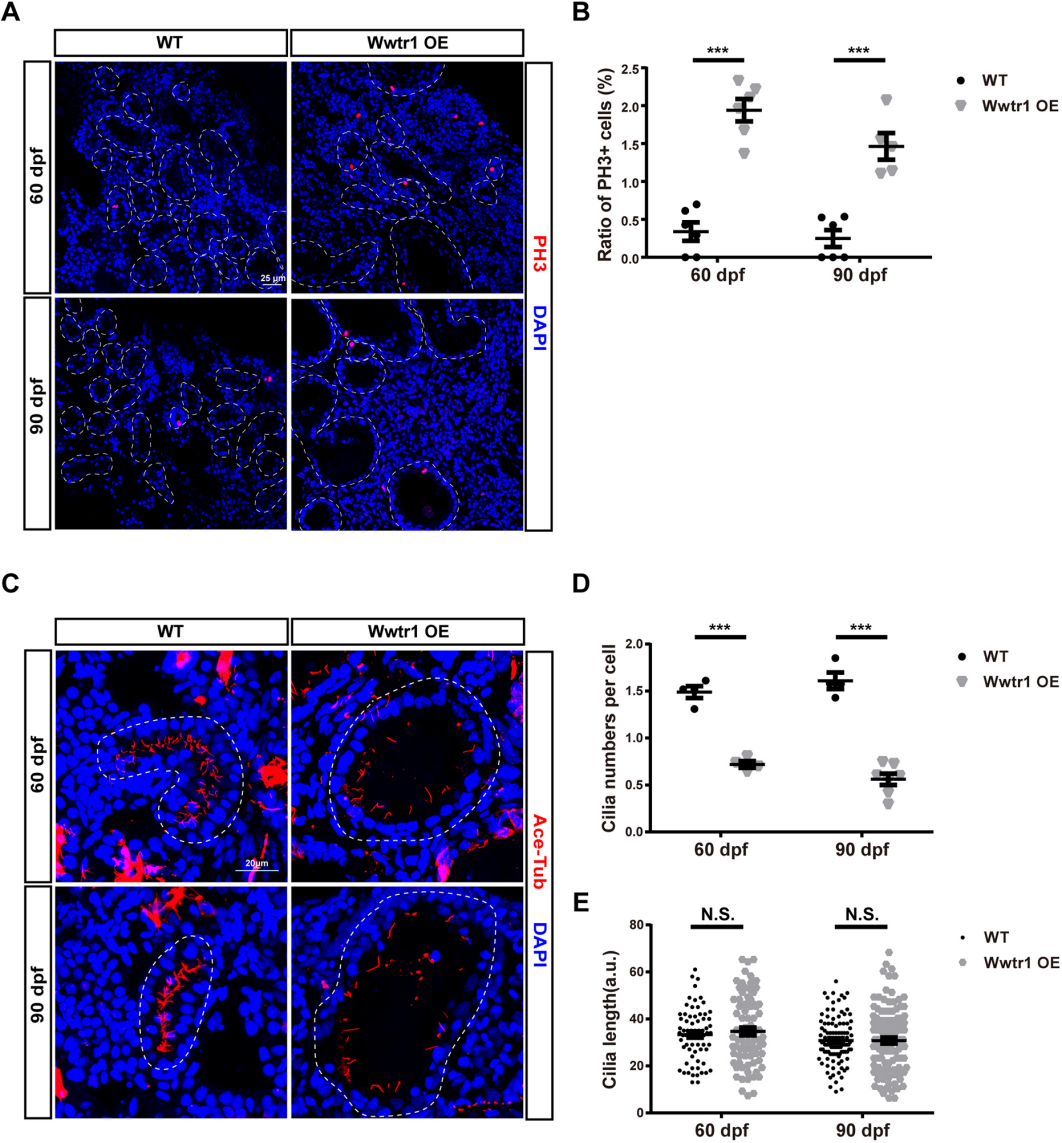
**Wwtr1 OE fish exhibit hyperproliferation of renal tubular epithelial cells and fewer cilia in renal tubules.** (A) Immunofluorescent staining of cryostat sections of kidneys in adult Wwtr1 OE and WT fish at 60 dpf and 90 dpf labeling PH3 (red) and DAPI (blue). Dashed line loops represent renal tubules. Scale bar: 25 μm. (B) Statistical analysis of the ratio of PH3^+^ cells in the renal tubules of Wwtr1 OE and WT fish [***P<0.001 by unpaired two-tailed Student's *t*-test, mean±s.e.m., *n*=6 WT (60 dpf), *n*=6 Wwtr1 OE (60 dpf), *n*=6 WT (90 dpf), *n*=5 Wwtr1 OE (90 dpf)]. (C) Immunofluorescent staining of acetylated tubulin (red) and DAPI (blue) in cryostat sections of kidneys of adult Wwtr1 OE and WT fish at 60 dpf and 90 dpf. *Z* projection of 0.5 μm optical section stacks. Dashed line loops represent renal tubules. Scale bar: 20 μm. (D) Statistical analysis of cilia numbers in renal tubules of Wwtr1 OE and WT fish. The cilia numbers in the whole renal tubules were counted and divided by the cell numbers [***P<0.001 by unpaired two-tailed Student's *t*-test, mean±s.e.m., *n*=4 WT (60 dpf), *n*=4 Wwtr1 OE (60 dpf), *n*=4 WT (90 dpf), *n*=7 Wwtr1 OE (90 dpf)]. (E) Statistical analysis of cilia lengths in renal tubules of Wwtr1 OE and WT fish. N.S., not significant by unpaired two-tailed Student's *t*-test, mean±s.e.m. [*n*=70 WT (60 dpf), *n*=78 Wwtr1 OE (60 dpf), *n*=105 WT (90 dpf), *n*=112 Wwtr1 OE (90 dpf)].

The Hippo signaling pathway has long been studied for its roles in organ size control through regulating cell proliferation. In mammals, cyst formation has been found to be associated with increased cell proliferation in PKD individuals (Nadasdy et al., 1995). To address this issue, we examined cell proliferation rate in Wwtr1 OE fish. Kidneys of Wwtr1 OE and WT fish were sampled for cryostat sectioning and immunostaining with anti-PH3 labeling phospho-histone H3 (a mitosis marker) to demonstrate cell proliferation (Fig. 7A). A significantly higher proliferation rate in tubular epithelial cells was observed in Wwtr1 OE fish compared to WT controls at both 60 dpf and 90 dpf (Fig. 7B).

### Cilia defects in the kidney of Wwtr1 OE fish

Studies have shown that cilia play important roles in renal cyst formation, and dysfunction of cilia has been associated with the pathology of PKD (Ma et al., 2017). The MST1/2-SAV1 complex was reported to promote ciliogenesis, and the loss of MST1/MST2 or activation of YAP1/WWTR1 mediated cilium disassembly in mammalian cell lines (Kim et al., 2015, 2014). To investigate whether Wwtr1 OE fish exhibited abnormalities in renal cilia, we performed cryostat sectioning and immunostaining with anti-ace-Tub to label cilia in the kidney at 60 dpf and 90 dpf. Maximal intensity projections of *z*-stack series were obtained to analyze the cilia in the kidney. In the kidney of WT controls, the cilia in the kidney were derived from epithelial cells and stretched to the lumen of renal tubules. As zebrafish renal tubules have both single ciliated cells and multiciliated cells, we counted the total numbers of cilia in renal tubules and divided them by total cell numbers. Significantly fewer cilia per cell were observed in the renal cysts of Wwtr1 OE fish compared to those of WT controls (Fig. 7C,D). However, the cilia length did not show obvious difference between Wwtr1 OE fish and WT controls (Fig. 7E).

### Stk3-deficient zebrafish is a potential PKD model for drug development

Zebrafish is widely used as a vertebrate model in drug development. In this study, we found that *stk3*^−/−^ mutants exhibited renal tubule dilation and glomerular cyst formation, which was mediated through Wwtr1 activation, at least partially. Furthermore, Wwtr1 OE fish exhibited PKD phenotypes, which were similar to those in mammals. To evaluate whether *stk3*^−/−^ mutants could be used as a PKD model for drug development, we tested the sensitivity of *stk3*^−/−^ mutants to different drugs. As *stk3*^−/−^ mutants exhibited edema between 12 dpf and 18 dpf, all treatments were initiated at 8 dpf and ended at 20 dpf. None of the drugs in our study had obvious effects on edema onset in *stk3*^+/+^ controls.

**Fig. 8.**
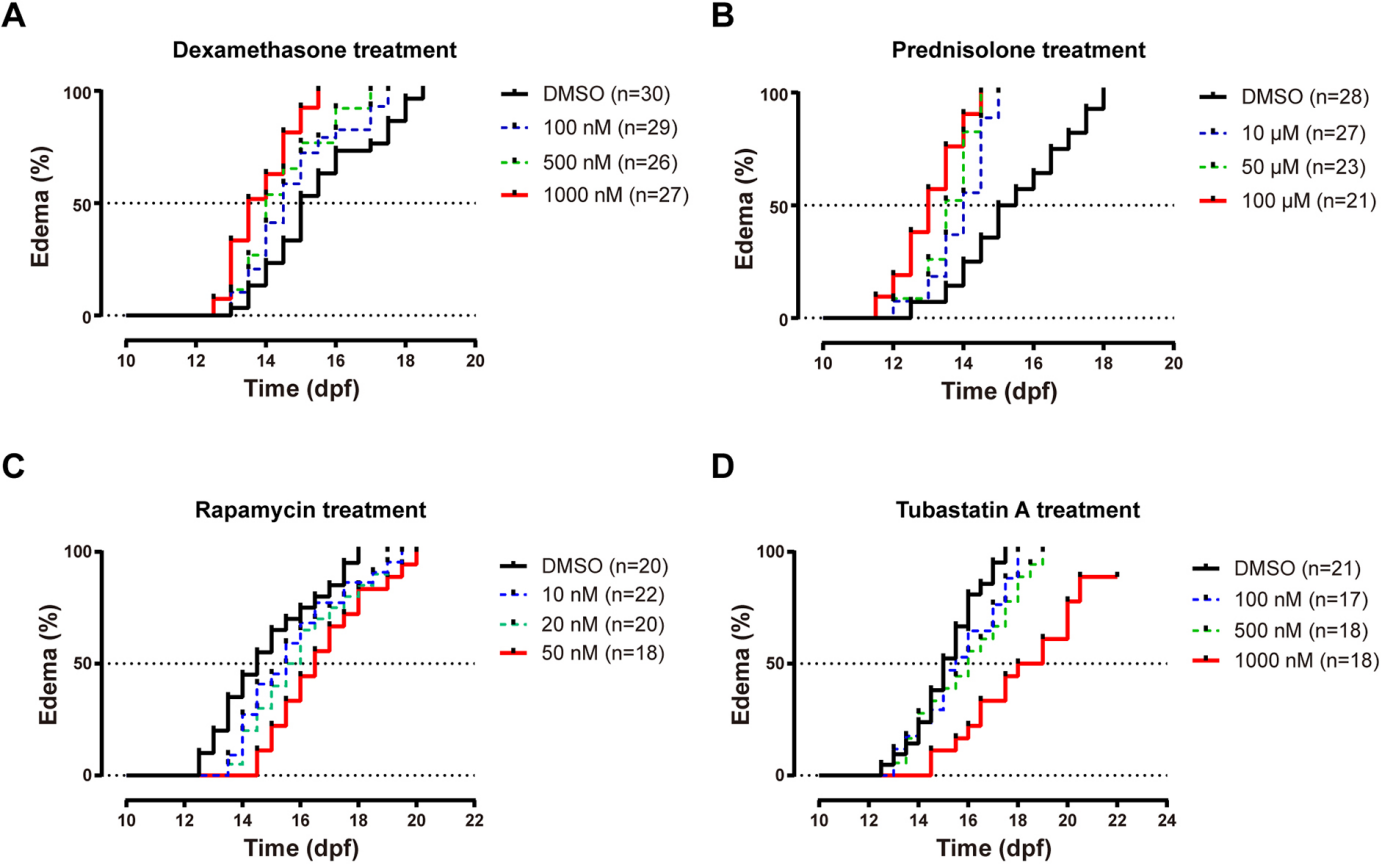
**Treatment of Stk3-deficient mutant zebrafish for edema.** None of the drugs in the study had obvious effects on edema onset in *stk3^+/+^* controls. (A) Dose response of dexamethasone-induced early onset of the edema phenotype in *stk3*^−/−^ larvae. The median times of onset of edema were 14.5 dpf (100 nM), 14.0 dpf (500 nM) and 13.5 dpf (1000 nM) compared to 15.0 dpf in DMSO control. Statistical analysis was performed using log-rank (Mantel–Cox) test (*P*<0.001). (B) Dose response of prednisolone-induced early onset of the edema phenotype in *stk3*^−/−^ larvae. The median times of onset of edema were 14.0 dpf (10 μM), 13.5 dpf (50 μM) and 13.0 dpf (100 μM) compared to 15.0 dpf in DMSO control. Statistical analysis was performed using log-rank (Mantel–Cox) test (*P*<0.001). (C) Delay of edema onset by rapamycin treatment. The median times of onset of edema were 15.5 dpf (10 nM), 15.75 dpf (20 nM) and 16.5 dpf (50 nM) compared to 14.5 dpf in DMSO control. Statistical analysis was performed using log-rank (Mantel–Cox) test (*P*<0.05). (D) Delay of edema onset by tubastatin A treatment. The median onset points were 15.5 dpf (100 nM), 16.0 dpf (500 nM) and 18.5 dpf (1000 nM) compared to 15.0 dpf in DMSO control. Statistical analysis was performed using log-rank (Mantel–Cox) test (*P*<0.001).

Previous studies have reported that corticosteroids can induce PKD in mammalian models (McDonald et al., 1990). Based on this, we tested whether *stk3*^−/−^ mutants were sensitive to treatments with corticosteroid agonists, dexamethasone and prednisolone, and the doses of treatments were as used in a previous study (Jobst-Schwan et al., 2019). The results showed that dexamethasone treatment aggravated the edema phenotype in *stk3*^−/−^ mutants in a dose-dependent manner. The median time of onset of edema was 15.0 dpf in the dimethyl sulfoxide (DMSO) group, and 14.5, 14.0 and 13.5 dpf in the 100, 500 and 1000 nM dexamethasone treatment groups, respectively (Fig. 8A). Similarly, prednisolone treatment also aggravated the edema phenotype in *stk3*^−/−^ mutants. The median time of onset of edema was 15.3 dpf in the DMSO group, and 14.0, 13.5 and 13.0 dpf in the 10, 50 and 100 μM prednisolone treatment groups, respectively (Fig. 8B). These results suggested that *stk3*^−/−^ mutants were sensitive to corticosteroid treatments.

The mTOR pathway has been reported to participate in cyst growth in mammals, and inhibition of the mTOR signaling reversed the renal cystogenesis (Shillingford et al., 2006; Tao et al., 2005; Wahl et al., 2006). To test whether inhibition of the mTOR pathway has similar effects on renal cystogenesis in the zebrafish, we treated fish with rapamycin, the mTOR pathway inhibitor, and the doses of treatments were as used in a previous study (Yin et al., 2016). As expected, rapamycin treatment significantly delayed the onset of edema in the *stk3*^−/−^ mutants. The median time of onset of edema was 14.5 dpf in the DMSO group, and 15.5, 15.75 and 16.5 dpf in the 10, 20 and 50 nM rapamycin treatment groups, respectively (Fig. 8C).

Histone deacetylase 6 (HDAC6) has also been reported to be involved in renal cyst formation in mammals (Ke et al., 2018). To test whether inhibition of HDAC6 reduces cystic cell proliferation and cyst growth, we treated fish with tubastatin A, an HDAC6 inhibitor. As expected, tubastatin A treatment significantly delayed the onset of edema in the *stk3*^−/−^ mutants. The median time of onset of edema was 15 dpf in the DMSO group, and 15.5, 16 and 18.5 dpf in the 100, 500 and 1000 nM tubastatin A treatment groups, respectively (Fig. 8D).

## DISCUSSION

In the past decade, gene-editing tools, especially transcription activator-like effector nucleases (TALENs) and CRISPR/Cas9, have been widely used for studying gene functions in zebrafish. Several groups have generated mutants related to the Hippo signaling pathway focusing on different functions, including early embryonic development (Kimelman et al., 2017), liver development (Yi et al., 2018), eye development (Miesfeld et al., 2015), blood vessel development (Astone et al., 2018; Nakajima et al., 2017), micropyle formation (Dingare et al., 2018; Yi et al., 2019) and cardiac wall maturation (Lai et al., 2018). However, the function of the Hippo signaling pathway in the zebrafish renal system has not been studied using a gene-knockout approach.

### Roles of the Hippo signaling pathway in zebrafish renal development

In *Drosophila*, disruption of the Hippo pathway mainly led to hyperproliferation and enlarged organ size, with little information available on renal development (Harvey et al., 2003; Huang et al., 2005; Justice et al., 1995). In a mouse model, podocyte-specific knockout of *Yap1* induced proteinuria and focal segmental glomerulosclerosis (Schwartzman et al., 2016). Deletion of *Yap1* in mouse cap mesenchyme led to impaired nephron induction and neonatal death (Reginensi et al., 2013). Furthermore, mice with nephric duct-specific deletion of *Yap1* exhibited defects in ureter-bladder junction (Reginensi et al., 2015), and renal tubule-specific *Mst1/2* deletion induced hyperproliferation of renal tubular epithelial cells and renal fibrosis (Xu et al., 2020).

In zebrafish, some studies have been published concerning the roles of the Hippo signaling pathway in pronephric development. Morpholino-mediated knockdown of *wwtr1* in zebrafish induced cystic dilatation of the pronephric tubules and changed their proximodistal patterning (Zhang et al., 2015). Also, Scrib was found to regulate renal cyst formation through Yap1, and either knockdown or overexpression of Yap1 induced cyst formation in zebrafish (Skouloudaki et al., 2009; Xu et al., 2018). In another study, morpholino knockdown of Yap1 in zebrafish resulted in pronephric cyst formation with ciliary defects (He et al., 2015). Although these studies provided some information concerning the involvement of the Hippo signaling pathway in renal development, they were mostly based on a morpholino-mediated knockdown approach. Morpholino knockdown was previously the most commonly used reverse genetics approach in zebrafish, owing to its efficiency and convenience, prior to the emergence of the genome-editing approaches (i.e. TALEN and CRISPR/Cas9). However, this approach has some shortages: the effects of morpholinos cannot last for longer than 5 days and are not heritable. Furthermore, recent studies have reported that ∼80% of morphant phenotypes were not observed in knockout mutants generated with a TALEN or CRISPR/Cas9 system (Kok et al., 2015). Despite this, morpholino-based gene knockdown is still being used as an ancillary tool for knockout approaches (El-Brolosy et al., 2019; Ma et al., 2019; Rossi et al., 2015).

In this study, we induced both gene knockout and gene knockdown through the CRISPR/Cas9 system. Our knockdown results in *yap1* and *wwtr1* crispants were consistent with previous studies (He et al., 2015; Zhang et al., 2015). Because the phenotypes of *yap1* and *wwtr1* crispants could be rescued by both *yap1* and *wwtr1* mRNAs, we believe that the CRISPR/Cas9-mediated knockdown was specific, without off-target effects. Interestingly, although the double mutant of *yap1* and *wwtr1* (*yap1*^−/−^*;wwtr1*^−/−^) showed embryonic lethality, demonstrating functional importance of the Hippo pathway in development, single knockout mutants of *yap1* and *wwtr1* did not exhibit pronephros defects as seen in their crispants. Examination of *yap1* and *wwtr1* double mutants suggested functional compensation between Yap1 and Wwtr1 in pronephros development. The loss of Wwtr1 could be fully compensated by both *yap1*^+/+^ and *yap*1^+/−^, whereas the loss of Yap1 could be rescued fully by *wwtr1*^+/+^ but only partially by *wwtr*1^+/−^, suggesting a gene-dose effect for *wwtr1*. The *yap1*^−/−^*;wwtr1*^+/−^ fish exhibited formation of pronephric cysts, which is similar to observations in *yap1* and *wwtr1* crispants. The functional compensation or redundancy revealed by *yap1* and *wwtr1* double mutants can well explain the lack of phenotypes in *yap1* and *wwtr1* single mutants, but not the phenotypes of their crispants. Such discrepancy between knockdowns and knockouts has been reported in a variety of organisms especially zebrafish (Rossi et al., 2015), but there is no clear explanation for the mechanism at this moment. Genetic compensation in response to gene mutations has been proposed for the reduced penetrance of mutant genes, which is a widespread phenomenon observed in a variety of organisms from yeast to humans (El-Brolosy and Stainier, 2017). Although the exact mechanism underlying genetic compensation remains poorly understood, two possibilities have been proposed: DNA damage in the mutant gene and mutated mRNA transcribed from the mutant gene. The DNA damage or lesion in the mutant may induce global chromatin reorganization, and the mutant mRNA may trigger mRNA decay pathways to produce RNA fragments, both of which may induce transcriptional adaptation response that results in compensation of the lost gene functions. Because the triggers of both mechanisms work upstream of protein translation, such genetic compensation occurs in knockouts, but not knockdowns (El-Brolosy et al., 2019). A recent study in zebrafish showed that the mutant of the *slc25a46* gene showed no phenotype whereas its crispant displayed severe disease symptoms. The discrepancy was due to genetic compensation that did not involve mRNA decay (Buglo et al., 2020). This observation was similar to our results on *yap1* and *wwtr1* mutants and crispants. Although we do not have evidence for the exact mechanism underlying genetic compensation in *yap1* and *wwtr1* single knockouts, changed expression of *yap1* and *wwtr1* is likely part of the transcriptional adaptation response for each other, as suggested by the double mutants.

For renal cystogenesis, we found that *stk3*^−/−^ mutants exhibited pronephric dysfunctions including renal glomerular cyst formation and tubule dilation, suggesting an important role for Stk3 in zebrafish renal development. Further evidence showed that Wwtr1, but not Yap1, was involved in phenotypic development of the *stk3*^−/−^ mutants. Wwtr1 OE fish phenocopied *stk3*^−/−^ mutants at larval stage and developed PKD symptoms at adult stage. Together, our findings suggest that loss of Stk3 in zebrafish induced pronephric dysfunctions through activation of Wwtr1 instead of Yap1, and these dysfunctions resulted in the symptoms of early-stage PKD.

### Roles of the Hippo signaling pathway in PKD in mammals and zebrafish

The Hippo signaling pathway has been implicated in renal diseases including PKD. In human ADPKD patients, strong nuclear YAP1 accumulation was observed in the cyst-lining cells (Happé et al., 2011). Gene set enrichment analysis revealed upregulation of YAP1 target genes in human ADPKD samples (Cai et al., 2018). In mice, deletion of *Wwtr1* resulted in PKD (Hossain et al., 2007; Makita et al., 2008). A recent study demonstrated that YAP1 was the direct downstream target of PKD1 mutations in ADPKD patients, and YAP1 was found to accumulate in nuclei of the tubular epithelial cells in a *Pkd1* knockout mouse (Cai et al., 2018). Loss of *Yap1*/*Wwtr1* dramatically reduced cyst formation induced by *Pkd1* deletion (Cai et al., 2018; Lee et al., 2020). As an upstream scaffold protein, kidney-specific deletion of *Sav1* in mice induced hyperproliferation of renal tubular epithelial cells and cyst formation through activating YAP1 (Kai et al., 2016). These studies provided solid evidence for important roles of the Hippo signaling pathway in renal cyst formation.

In the present study on zebrafish model, global deletion of *stk3* also resulted in the development of PKD. We further demonstrated the involvement of Wwtr1, but not Yap1, in cystogenesis. Although global deletion of *wwtr1* did not induce PKD in zebrafish, overexpression of its active form caused severe renal cyst formation. Although the Hippo signaling pathway is clearly involved in PKD of both mammals and zebrafish, the regulation of the pathway seemed different in these models. In mammals, YAP1 and WWTR1 seem to function redundantly in cystogenesis. However, it was the activation of Wwtr1, but not Yap1, that served as the main effector for cystogenesis in zebrafish. Despite this difference, evidence of the lost function of the Hippo signaling pathway in PKD development is obviously similar between mammals and zebrafish, including cyst formation in renal tubules, hyperproliferation of renal tubular epithelial cells and cilia dysfunction. Increased proliferation of renal tubular epithelial cells seems to be the major cause of renal cyst formation (Nadasdy et al., 1995). As many receptors are located on the cilia, cilia dysfunction can impair signal transduction and cell homeostasis, which can accelerate cyst formation (Ma et al., 2017). Together, our results provide additional evidence for the function of the Hippo signaling pathway in renal cyst formation in vertebrates.

### Novel zebrafish models for PKD

The zebrafish has become a popular model for studying human genetic diseases. Zebrafish and human genomes have high orthology (Howe et al., 2013). High fecundity, small body size, *ex utero* development, transparent embryos and low maintenance cost make zebrafish an excellent model for high-throughput screening. Recently, the application of gene-editing tools in zebrafish has enabled us to target different genes for disease modeling (Zhu and Ge, 2018).

Studies so far on PKD modeling in zebrafish have adopted a few zebrafish lines that form renal cysts in pronephros at embryonic stage (Gehrig et al., 2018; Mangos et al., 2010; Obara et al., 2006; Poureetezadi and Wingert, 2016). In the present study, we generated a Stk3-deficient zebrafish line that could serve as a potential model for PKD. In particular, this fish mutant line could be used as a tool for drug development, as demonstrated by our pharmacological experiments. We demonstrated that the median time of onset of edema was a reliable criterion to assess drug effects. With this model, we can also investigate the possible pharmacological mechanisms underlying drug actions. Our results showed that treatment of the *stk3*^−/−^ mutant with corticosteroids aggravated the edema phenotype, which might be due to changed threshold of corticosteroid tolerance in the *stk3* mutant. Previous studies reported that the mTOR pathway can be activated by YAP/TAZ in the kidney (Gui et al., 2018). We showed that inhibition of the mTOR pathway can alleviate the edema phenotype in the *stk3*^−/−^ mutant. Treatment with tubastatin A (an HDAC6 inhibitor) delayed the onset of edema in the *stk3*^−/−^ mutant. As the *stk3*^−/−^ mutant developed edema through the activation of Wwtr1, which led to cell proliferation, inhibition of proliferation by the HDAC6 inhibitor reduced cystic formation.

The Wwtr1 OE line we created was a novel zebrafish line that exhibited PKD phenotypes in mesonephros at adult stage. This transgenic line could serve as a model to investigate the progression of PKD. In the future, we may cross the Wwtr1 OE line with the transparent *casper* line (White et al., 2008) and lines with kidney-specific expression of fluorescent proteins (such as *cdh17:mCherry*) (Diep et al., 2011) to generate PKD models with transparent body and kidney-specific fluorescence, which may enable us to monitor the progression of PKD *in vivo*.

In summary, we have provided solid evidence, using a genetic approach, for involvement of the Hippo signaling pathway in zebrafish renal development. Furthermore, we established two novel zebrafish PKD models for both embryonic or larval stage and adults, which have great potential not only for investigating the disease and its progression during development, but also for drug screening and therapy development.

## MATERIALS AND METHODS

### Fish and maintenance

All the experiments in this study were performed on AB strain zebrafish. The larvae were first raised in a Forma Environmental Chamber (Model 3949; Thermo Fisher Scientific) and then maintained in a ZebTEC Multilinking Rack Zebrafish System (Tecniplast, Buguggiate, Italy), with the photocycle of 14 h light and 10 h dark. The temperature, pH and conductivity of the system water were 28±1°C, 7.5 and 400 µS/cm, respectively. All experiments were performed according to the protocols approved by the Research Ethics Panel of the University of Macau.

### Establishment of mutant lines

The CRISPR/Cas9 system was applied to generate the zebrafish mutant lines according to the protocols reported previously (Lau et al., 2016). The targets were designed with ZIFIT Targeter (http://zifit.partners.org/zifit). Oligonucleotides synthesized were annealed and inserted into the pDR274 vector. The sgRNAs and Cas9 RNA were prepared using MEGAscript T7 and mMESSAGE mMACHINE SP6 kits (Life Technologies, Carlsbad, CA, USA), respectively. The mixture (4.6 nl) of sgRNA (20 ng/μl) and Cas9 mRNA (200 ng/μl) was co-injected into one-cell-stage embryos with a Nanoject system (Drummond, Broomall, PA, USA) to generate the F0 mutant fish. F0 fish were raised and those carrying mutations were outcrossed with WT fish to obtain F1 fish. The oligonucleotides used in this study are listed in Table S1.

### Gene knockdown using CRISPR/Cas9 system and mRNA rescue

The CRISPR/Cas9 system was applied to knock down the target genes. The target site design and mRNA preparation were performed as described above. The mixture (4.6 nl) of sgRNA (20 ng/μl) and Cas9 mRNA (200 ng/μl) was co-injected into one-cell-stage embryos with the Drummond Nanoject system to generate the knockdown crispants. To rescue the phenotypes of crispants, *yap1* mRNA (100 pg) or *wwtr1* mRNA (24 pg) was prepared using mMESSAGE mMACHINE SP6 kits and co-injected with sgRNA and Cas9 mRNA. The amounts of mRNA injected were based on previous studies (He et al., 2015; Zhang et al., 2015).

### Establishment of transgenic lines

The CDS of *yap1* was cloned from zebrafish cDNA and a mutation was introduced using overlap PCR, which converted the 87th amino acid from serine to alanine (Yap1^S87A^). This sequence was inserted into the pSK-GFP (Tol2-CMV-GFP-pA-CMV-MCS-pA-Tol2) vectors using a Gibson Assembly kit (NEB, Ipswich, MA, USA). The mixture (4.6 nl) of Tol2 transposase mRNA (50 ng/μl) and Wwtr1 overexpression vector (20 ng/μl) was co-injected into one-cell-stage embryos to generate the Yap1 OE line. The positive embryos were screened by monitoring the GFP signals. The Wwtr1 OE line was generated with the same approach.

### Sampling and histological analysis

Zebrafish larvae and adult fish were anesthetized by MS222 (Sigma-Aldrich, St Louis, MO, USA) and imaged with a SMZ18 stereomicroscope (Nikon, Tokyo, Japan) or digital camera (Canon EOS 700D). Then, the whole fish or dissected kidneys were fixed in Bouin's fixative for 24 h before processing. After standard histological processing, the samples were embedded in paraffin and sectioned at 5 μm for H&E staining. The slides were viewed under an ECLIPSE Ni-U microscope (Nikon) and imaged with a Digit Sight DS-Fi2 digital camera (Nikon).

### Immunofluorescence

For whole-mount immunofluorescence, zebrafish larvae were fixed in 4% paraformaldehyde (PFA) overnight at 4°C and processed for immunostaining as described previously (Hammond-Weinberger and ZeRuth, 2020).

Zebrafish larvae or kidneys of adult fish were fixed in 4% PFA overnight at 4°C and then incubated in 30% sucrose overnight. The samples were embedded in optimal cutting temperature (OCT) medium and sectioned at 10 µm on a CM5030 cryostat (Leica, Wetzlar, Germany). After being air-dried overnight, the slides were washed with PBS for 3×5 min. The slides were placed in a humid chamber and incubated with block buffer (5% normal horse serum, 0.1% Triton X-100 in 1× PBS) overnight at 4°C. The following day, the slides were incubated with primary antibodies diluted with block buffer overnight at 4°C, incubated with secondary antibodies for 2 h and mounted with ProLong Gold Antifade Reagent (Invitrogen). Images were acquired using a Nikon A1MP+ fluorescence confocal microscope and analyzed with Fiji software (ImageJ). The antibodies used are listed in Table S2.

### Western blotting

For sample preparation, zebrafish embryos at 5 dpf were homogenized and lysed in RIPA buffer (Thermo Fisher Scientific) on ice. The supernatant was collected and heated at 95°C for 10 min with loading buffer. The supernatant was used for western blot analysis according to the standard protocol (Li and Ge, 2011). The antibodies used are listed in Table S2.

### Drug treatments

Embryos were obtained with *stk3*^+/−^ in-cross and distributed randomly to different treatment groups. Drugs or vehicle control were added to fish water at 8 dpf and changed every other day. All treatments were initiated at 8 dpf and ended at 20 dpf. The larvae were monitored twice a day, and individuals with edema were recorded and genotyped. The remaining individuals were all genotyped at the end of the experiment.

Verteporfin, dexamethasone, prednisolone, rapamycin and tubastatin A were all purchased from Sigma-Aldrich and dissolved in DMSO as stocks. All treatment and vehicle control groups contained 0.001% DMSO.

### Data analysis

Kaplan–Meier plots for the onset of edema and survival were analyzed using the log-rank (Mantel–Cox) test. All other values in this study were expressed as mean±s.e.m., and statistical significance was analyzed by one-way ANOVA or unpaired Student's *t*-test using Prism (GraphPad, San Diego, CA).

## Supplementary Material

Supplementary information
