# Supplementary material for: Novel zebrafish polycystic kidney disease models reveal functions of the Hippo pathway in renal cystogenesis
Source: Dis Model Mech. 2021 Nov 9;14(11):dmm049027. doi: 10.1242/dmm.049027 (PMC8592019; doi:10.1242/dmm.049027)
Supplement: Supplementary information [file dmm-14-049027-s1.pdf]

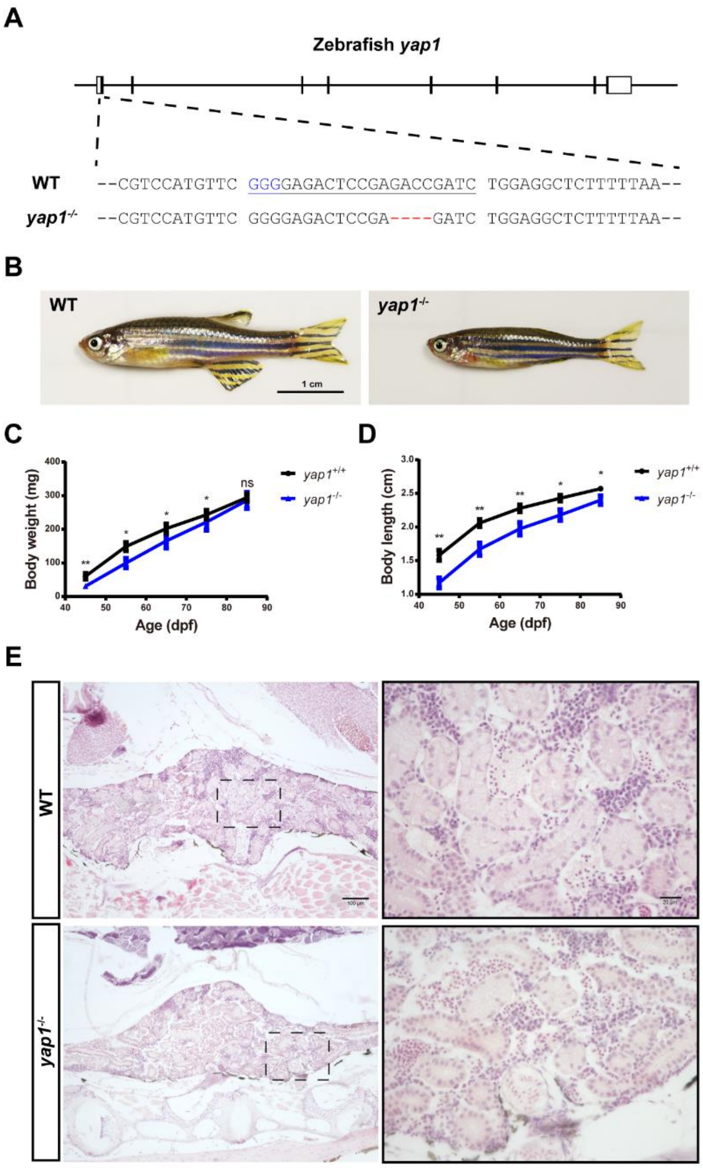

Fig. S1. Yap1 mutants (*yap1*$^{-/-}$) suffered from growth retardation during juvenile but developed normal kidneys. (A) Schematic diagram of *yap1* gene structure and CRISPR-induced mutation. The CRISPR site was designed to target the 1st exon. A mutant line with 4-bp deletion was generated for phenotype analysis. Solid boxes indicate coding regions and open boxes indicate untranslated regions. The underlined sequence shows the CRISPR site. The PAM sequence is shown in blue, and the altered sequence is shown in red. (B) Morphology of adult fish at 65 dpf. The mutant fish (*yap1*$^{-/-}$) exhibited smaller body sizes than WT fish. Scale bar: 1 cm. (C) Statistical analysis of body weights of *yap1*$^{-/-}$ fish and WT siblings from 45 to 85 dpf. * P <0.05; ** P <0.01; ns, not significant by unpaired two-tailed Student's *t*-test, mean±s.e.m. (n=10). (D) Statistical analysis of body lengths of *yap1*$^{-/-}$ fish and WT siblings from 45 to 85 dpf. * P <0.05; ** P <0.01 by unpaired two-tailed Student's *t*-test, mean±s.e.m. (n=10). (E) H&E staining of paraffin sections in *yap1*$^{-/-}$ fish and WT siblings at 90 dpf. Scale bars: 100 µm and 20 µm, respectively.

Disease Models & Mechanisms • Supplementary information

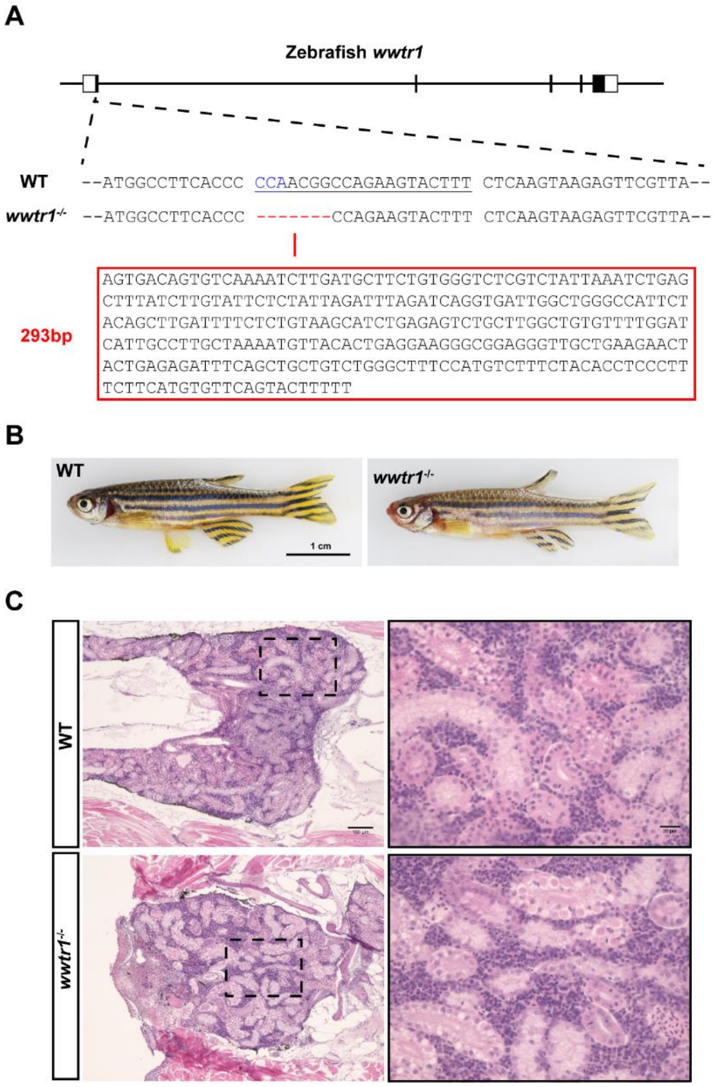

Fig. S2. Wwtr1 mutants (*wwtr1*$^{-/-}$) developed normal kidneys. (A) Schematic diagram of *wwtr1* gene structure and CRISPR-induced mutation. The CRISPR site was designed to target the 1st exon. A mutant line with 7-bp deletion and 293-bp insertion was generated for phenotype analysis. Solid boxes indicate coding regions and open boxes indicate untranslated regions. The underlined sequence shows the CRISPR site. The PAM sequence is shown in blue, and the altered sequence is shown in red. (B) Morphology of *wwtr1*$^{-/-}$ fish and WT siblings at 90 dpf. Scale bar: 1 cm. (C) H&E staining of paraffin sections in *wwtr1*$^{-/-}$ fish and WT siblings at 90 dpf. Scale bars: 100 µm and 20 µm, respectively.

Disease Models & Mechanisms • Supplementary information

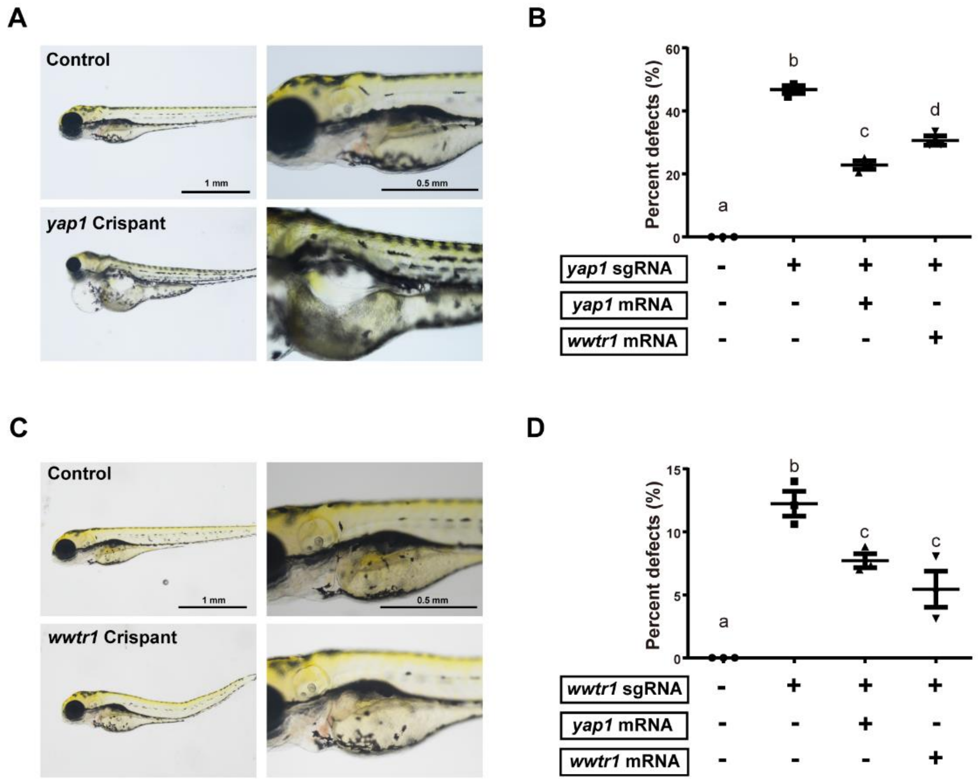

Fig. S3. Knockdown of *yap1* and *wwtr1* using CRISPR/Cas9 system. (A) Phenotypes of *yap1* crispants and control. The *yap1* crispants exhibited pericardial edema, pronephric cyst and curved body. Scale bars: 1 mm and 0.5 mm, respectively. (B) The mRNAs of *yap1* and *wwtr1* could both rescue the phenotypes of *yap1* crispants. Different letters indicate statistical significance by one-way ANOVA, mean±s.e.m. (n=3). (C) Phenotypes of *wwtr1* crispants and control. The *wwtr1* crispants exhibited pronephric cyst and curved body. Scale bars: 1 mm and 0.5 mm, respectively. (D) The mRNAs of *yap1* and *wwtr1* could both rescue the phenotypes of *wwtr1* crispants. Different letters indicate statistical significance by one-way ANOVA, mean±s.e.m. (n=3).

Disease Models & Mechanisms • Supplementary information

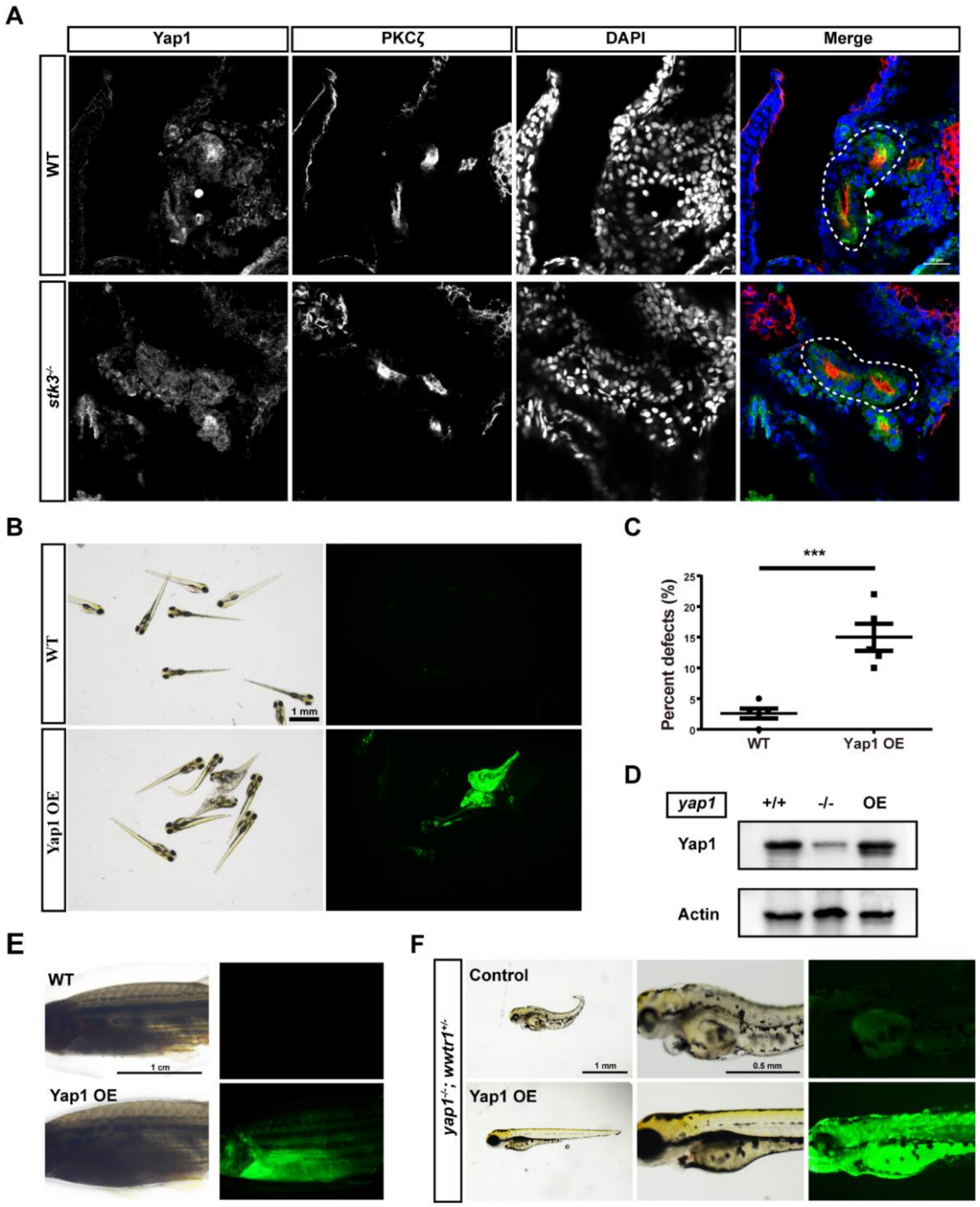

Fig. S4. Functional analysis of Yap1 in zebrafish kidneys. (A) Immunofluorescent staining of cryostat sections in *stk3*$^{-/-}$ larvae and WT siblings at 15 dpf labeling Yap1, PKCζ and DAPI. Merged images are shown with Yap1 staining in green, PKCζ in red and DAPI in blue. Dotted line loops represent renal tubules. Scale bar: 20 µm. (B) Morphology and fluorescent signal of larvae at 3 dpf. Scale bar: 1 mm. (C) Statistical analysis of embryonic defects in Yap1 OE fish and WT fish. *** P<0.001 by unpaired two-tailed Student's *t*-test, mean±s.e.m. (n=5). (D) Western blot for Yap1 in different genotypes. The weak band in *yap1*$^{-/-}$ was likely a non-specific reaction. (E) Morphology and fluorescent signal of Yap1 OE fish and WT controls at 60 dpf. Scale bar: 1 cm. (F) Yap1 OE could rescue the phenotype of *yap1*$^{-/-}$;*wwtr1*$^{+/-}$ fish. Scale bars: 1 mm and 0.5 mm, respectively.

Disease Models & Mechanisms • Supplementary information

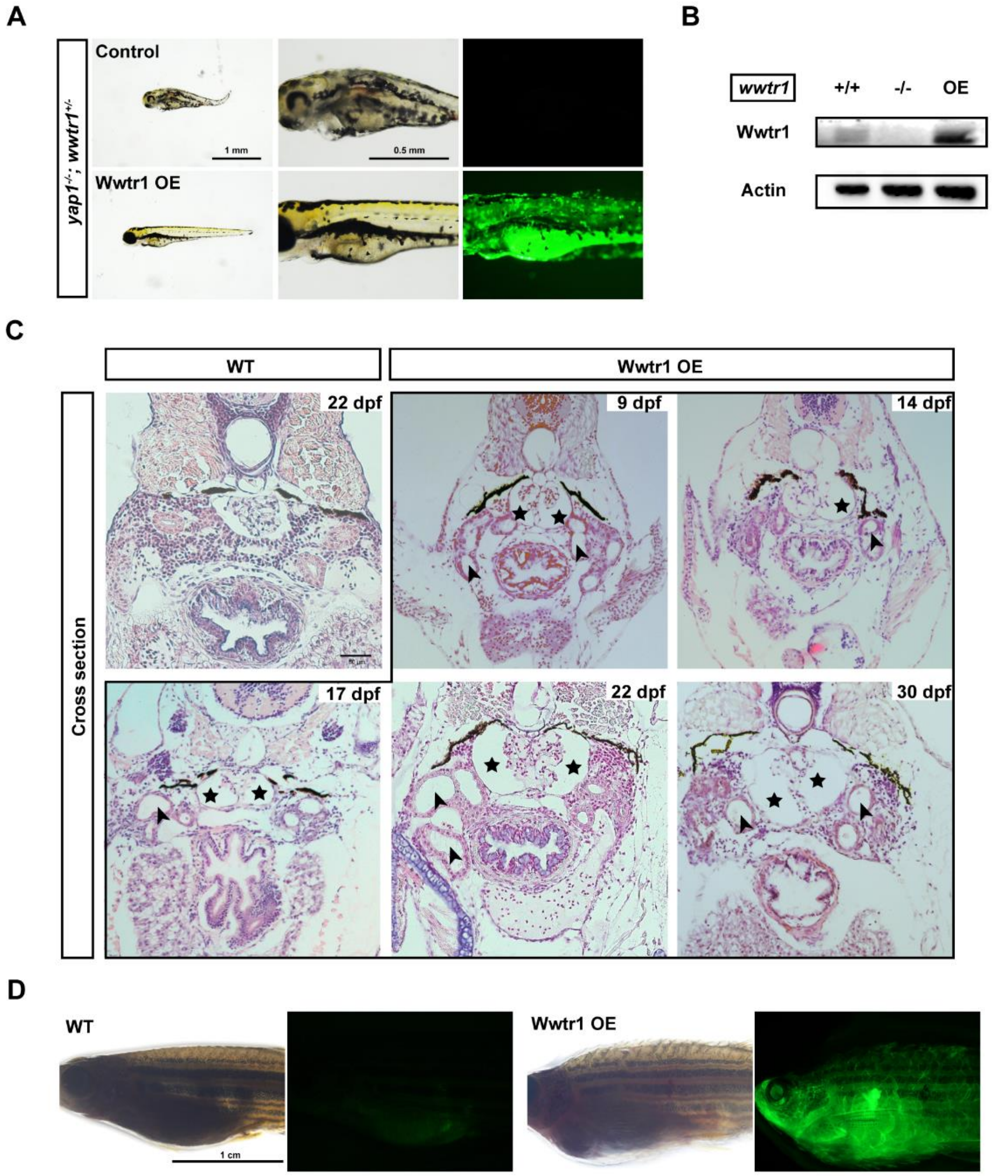

Fig. S5. Phenotype analysis of Wwtr1 OE fish. (A) Wwtr1 OE could rescue the phenotypes of *yap1*$^{-/-}$;*wwtr1*$^{+/-}$ fish. Scale bars: 1 mm and 0.5 mm, respectively. (B) Western blot for Wwtr1 in different genotypes. (C) H&E staining of paraffin cross sections in Wwtr1 OE larvae and WT controls at different time points. Asterisk: enlarged Bowman's space. Arrowhead: pronephric tubule dilation. Scale bar: 50 µm. (D) Morphology and fluorescent signal of Wwtr1 OE fish and WT controls at 65 dpf. Scale bar: 1 cm.

Disease Models & Mechanisms • Supplementary information

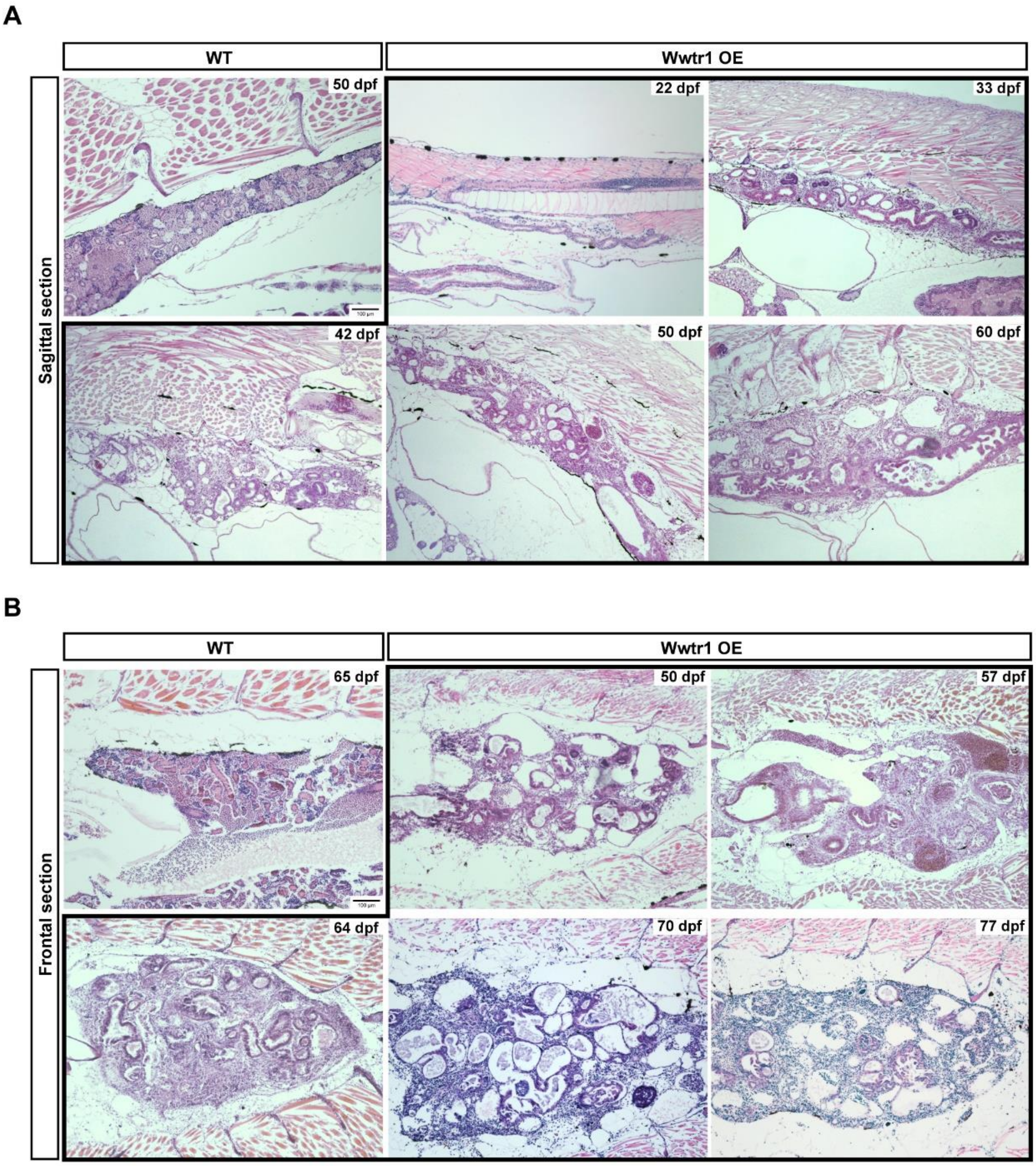

Fig. S6. Phenotype analysis of Wwtr1 OE fish (continued). (A) H&E staining of paraffin sagittal sections in Wwtr1 OE fish and WT controls at different time points. Scale bar: 100 µm. (B) H&E staining of paraffin frontal sections in Wwtr1 OE fish and WT controls at different time points. Scale bar: 100 µm.

Disease Models & Mechanisms • Supplementary information

Table S1. Oligonucleotides used in this study.

Gene name	Primer name	Primer sequence (5’ to 3’)	Application
stk3	3226	TAGGATGTATGTCTGCATACGG	sgRNA
	3227	AAACCCGTATGCAGACATACAT	
	3228	GTCTCTCGGCATCACATCCA	Genotyping
	3229	AGACTCCTCTCAGTGTCCTCC	
yap1	3220	TAGGGAGACTCCGAGACCGATC	sgRNA
	3221	AAACGATCGGTCTCGGAGTCTC	
	3222	CATGGATCCGAACCAGCACA	Genotyping
	3223	GGACGATGGTGTTTTTCGGG	
	4681	GGCGGCGTGAAGAATGAG	
	4682	TCGGGGAGACTCCGAGACCG	
	4683	TCGGGGAGACTCCGAGAGGC	
wwtr1	5796	TAGGTGTGGGAGTGGAGTCTCCCG	sgRNA
	5797	AAACCGGGAGACTCCACTCCCACA	
	5928	ACCAGTCCTGCGATGTG	Genotyping
	5929	GAATGTCCAGTAATAACGAAC	

Table S2. Primary antibodies used in this study

Antibody name	Source	Catalogue number	Dilutions
Anti-YAP1 antibody	Abcam	ab81183	1:200 (IF)<br>1:500 (WB)
PKC ζ Antibody (H-1)	Santa Cruz	sc-17781	1:50 (IF)
YAP/TAZ (D24E4) Rabbit mAb	Cell Signaling Technology	#8418	1:200 (IF)<br>1:500 (WB)
Phospho-Histone H3 (Ser10) Antibody	Cell Signaling Technology	#9701	1:500 (IF)
Monoclonal Anti-Tubulin, Acetylated antibody produced in mouse	Sigma-Aldrich	T6793	1:500 (IF)
β-Actin Antibody	Cell Signaling Technology	#4967	1:1000 (WB)

IF, immunofluorescence; WB, western blotting.
